# Selectivity and Safety Characterization of a Xanthine–Imidazothiazole Lead Structure: a Novel Tryptophan Hydroxylase Inhibitor of Peripheral Serotonin Synthesis

**DOI:** 10.1021/acsptsci.5c00043

**Published:** 2025-05-19

**Authors:** Radoslaw Wesolowski, Anja Schütz, Michael Lisurek, Marc Nazaré, Udo Heinemann, Dirk Pleimes, Michael Bader, Edgar Specker

**Affiliations:** † 28417 Trypto Therapeutics GmbH, Robert-Rössle Str. 10, Berlin 13125, Germany; ‡ Max-Delbrück-Center for Molecular Medicine in the Helmholtz Association (MDC), Robert-Rössle-Str. 10, Berlin-Buch 13125, Germany; § 27716 Chemical Biology Platform, Leibniz-Forschungsinstitut für Molekulare Pharmakologie (FMP), Robert-Rössle-Str. 10, Berlin-Buch 13125, Germany; ∥ German Center for Cardiovascular Research (DZHK), Partner Site Berlin, Potsdamer Str. 58, Berlin 10785, Germany; ⊥ Charité - Universitätsmedizin Berlin, Charitéplatz 1, Berlin 10117, Germany; # Experimental and Clinical Research Center, a cooperation between the Max-Delbrück-Center for Molecular Medicine in the Helmholtz Association and the Charité - Universitätsmedizin Berlin, Lindenberger Weg 80, Berlin-Buch 13125, Germany; ∇ University of Lübeck, Institute for Biology, Ratzeburger Allee 160, Lübeck 23562, Germany

**Keywords:** Serotonin, neurotransmitter, TPH1 Inhibitor, TPT-004

## Abstract

Serotonin (5-HT), a crucial neurotransmitter and peripheral mediator, regulates various physiological processes and is synthesized by tryptophan hydroxylase 1 (TPH1), the rate-limiting enzyme responsible for its production. 5-HT overproduction is implicated in multiple diseases, making TPH1 a promising therapeutic target. However, selectivity remains a challenge due to the structural similarity of TPH1 with other members of the aromatic amino acid hydroxylase (AAAH) family, including TPH2, phenylalanine hydroxylase (PAH), and tyrosine hydroxylase (TH). This study aimed to evaluate the selectivity and inhibitory potential of TPT-004, a novel TPH inhibitor, compared with Telotristat (LP778902) and its prodrug (LX1606). We developed high-throughput fluorescence assays to evaluate the inhibitory effects of the test compounds on TPH1, TPH2, PAH, and TH enzymes. TPT-004 demonstrated high selectivity for TPHs compared to LP778902 and LX1606. Structural analysis based on a detailed sequence alignment within the AAAH enzyme family, combined with cocrystal structures of TPH1 and TPH2 bound to different generations of inhibitors, enhances our understanding of the molecular basis of inhibitor binding and provides a framework for explaining TPT-004’s selectivity for TPHs. Selectivity profiling against 97 targets confirmed that TPT-004 showed minimal off-target interactions, underscoring its specificity. A dose–range finding (DRF) study in rats assessed the *in vivo* safety profile of TPT-004, showing no adverse effects on survival and body weight at doses up to 400 mg/kg/day. Hematology parameters remained normal, with only minor liver changes observed. These results highlight TPT-004’s potential as a selective and safe TPH inhibitor, offering a promising therapeutic option for serotonin-related disorders.

## Graphical abstract

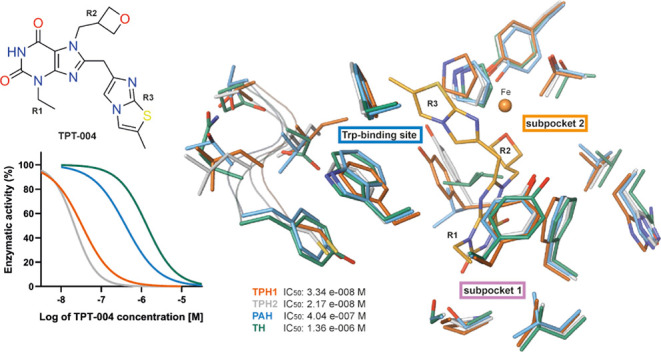

Serotonin (5-HT) is a potent neurotransmitter and a peripheral mediator. It plays a crucial role in modulating mood, cognition, and various physiological processes, such as sleep, appetite, and gut motility. Its excessive synthesis in the lower body by the rate-limiting enzyme tryptophan hydroxylase 1 (TPH1) is an underlying cause of pathogenesis in multiple diseases, such as carcinoid syndrome, pulmonary arterial hypertension, metabolic dysfunction-associated steatotic liver disease (MASLD), obesity, and fibrotic disorders.^1–7^ In recent years, there has been growing interest in understanding the molecular mechanisms and therapeutic potentials of targeting serotonin pathways to address these conditions.^8–10^ For that reason, TPH1 is considered an attractive therapeutic target to address the unmet medical needs in serotonin-related indications.^9,11,12^ TPH1 belongs to the iron- and pterin-dependent aromatic amino acid hydroxylase (AAAH) family, which also includes three other members: tryptophan hydroxylase 2 (TPH2; mostly expressed in brain tissue),^13^ phenylalanine hydroxylase (PAH),^14^ and tyrosine hydroxylase (TH).^15^ These enzymes share a conserved catalytic domain, which binds the essential cosubstrate tetrahydrobiopterin (BH_4_), iron, and their respective amino acid substrates.^16^ Due to the similar structure and highly conserved active sites across these enzymes, the TPH1 inhibitors (TPHi) intended for clinical use should present good target selectivity to avoid the risk of potential adverse effects by unspecific inhibition of TPH2, PAH, and TH. A popular strategy to circumvent the inhibition of brain-specific TPH2 is through a targeted drug design approach that prevents these inhibitors from crossing the blood-brain barrier. Most of the currently developed or clinically available tryptophan hydroxylase inhibitors target the enzyme’s active catalytic site, resulting in similar affinities for both TPH1 and TPH2. Despite previous efforts by Novartis to develop TPH inhibitors utilizing a novel allosteric site on TPH1, aimed at achieving selectivity over related aromatic amino acid hydroxylases, no progress has been reported since 2017.^17^

To date, there are no available sources comparing the inhibitory potentials of the available TPH inhibitors for all four AAAH members simultaneously. Moreover, the methodology described in available publications is based on different measurement methods (absorbance, fluorescence, HPLC, and scintillation readouts). Finally, the rationale for the concentrations of the substrate and cosubstrate used in those assays is often unclear, leading to a large variance in the experimental data and the reported half-maximal inhibitory concentration (IC_50_ values) across the publications.^17,18^ These inconsistencies highlight the need for standardized, high-throughput, and reliable assays to accurately assess the inhibitory effects of the potential therapeutic compounds. To overcome this limitation, we developed and optimized four independent AAAH assays (for TPH1, TPH2, PAH, and TH), intended for simple and reliable fluorescence measurements of the reaction products 5-HT and tyrosine in a 96-well plate format.^19,20^ The presented experimental setup is based on the unique Michaelis constant (the substrate concentration needed for half-maximum velocity, *K*_m_ values) for the main substrates (l-Trp or l-Phe or l-Tyr) and the cosubstrate tetrahydrobiopterin (BH_4_) determined separately for each hydroxylase, following the high-throughput screening (HTS) principles.

In our previous reports, we presented a novel class of xanthine–benzimidazoles, xanthine–imidazopyridines, and xanthine–imidazothiazoles TPH inhibitors, utilizing an active drug approach and an enhanced double-binding mode to target both catalytic subpockets (tryptophan and tetrahydrobiopterin) of TPH1, distinguishing them from existing TPH inhibitors.^21–24^ TPT-004, our lead compound, was designed based on the structure–activity relationship (SAR) approach to improve potency and selectivity compared to existing TPHi and minimize the penetration into the brain tissue. TPT-004 has previously demonstrated its therapeutic efficacy in the MC38 colorectal carcinoma model in mice as well as in the Sugen–Hypoxia model of pulmonary arterial hypertension in rats, making it an attractive drug development candidate.^22,25^

Therefore, one of the main objectives of the current study was to profile the inhibitory potential of TPT-004 in a battery of four AAAH enzymatic assays to determine its potency on the main target (TPH1) and selectivity toward highly similar off-target hydroxylases (TPH2, PAH, and TH) in comparison to clinically approved TPH inhibitors, Telotristat ethyl (LX1606; the prodrug form) and Telotristat (LP778902; the active metabolite).^26,27^ In addition, we evaluated the selectivity profile of TPT-004 against a broad panel (97) of common off-target receptors, enzymes, and ion channels using the Eurofins Diversity Panel. Finally, we conducted a dose–range finding (DRF) study in rats to evaluate the preliminary *in vivo* safety profile of TPT-004 and rule out any systemic effects of the subchronic treatment using high TPT-004 doses reaching 400 mg/kg per day, focusing on the survival, clinical signs, hematology, clinical chemistry, and histopathological assessment.

## Results

### Potency and Selectivity Profiling of TPT-004 in AAAH Assays

To determine the selectivity of TPT-004 toward the particular AAAH members, we established four independent enzymatic assays for each hydroxylase (recombinant human TPH1, TPH2, PAH, and TH) and optimized them for fluorescence measurements in a 96-well plate format. According to the principles of the HTS enzymatic assay development, for the identification of competitive inhibitors in a competition experiment that measures IC_50_ values, a substrate/cosubstrate concentration around the enzyme’s *K*_m_ value should be used.^28^ Table S1 shows the *K*_m_ values for the main substrates (l-Trp or l-Phe or l-Tyr) and cosubstrate (BH_4_) determined separately for each hydroxylase, and the conditions selected for the subsequent enzymatic assays. Besides evaluating TPT-004, we tested LP778902 (Telotristat) as a reference TPH inhibitor. Telotristat is the active metabolite of Telotristat ethyl (LX1606, brand name Xermelo®), the only clinically approved serotonin synthesis inhibitor for treating carcinoid syndrome.^26,27^

First, TPT-004, LP778902, and LX1606 were tested in the concentration range from 3 nM to 10 μM in the TPH1 assay (Figure 1A). All three compounds exhibited strong, dose-dependent inhibition of TPH1. The relative enzymatic activity of TPH1 after treatment with different concentrations of TPT-004, LP778902, and LX1606 is shown in Table S2. Based on the nonlinear regression model fitted to the experimental data, the IC_50_ of TPT-004 in the TPH1 enzymatic assay was determined to be 3.338 × 10^–08^ M, whereas the TPH1 IC_50_ of LP778902 was determined to be 5.92 × 10^–07^ M. The IC_50_ of LX1606 was determined to be 1.44 × 10^–06^ M. Similarly, TPT-004, LP778902, and LX1606 were tested in the concentration range from 3 nM to 10 μM in the TPH2 assay (Figure 1B), all demonstrating strong, dose-dependent inhibition of the TPH2 enzyme. The relative enzymatic activity of TPH2 after treatment with various concentrations of these compounds is shown in Table S3. For TPT-004, the TPH2 IC_50_ was determined to be 2.17 × 10^–08^ M, while for LP778902, the TPH2 IC_50_ was 5.86 × 10^–07^ M. The IC_50_ of LX1606 in the TPH2 enzymatic assay was determined to be 1.24 × 10^–06^ M.

**Figure 1:**
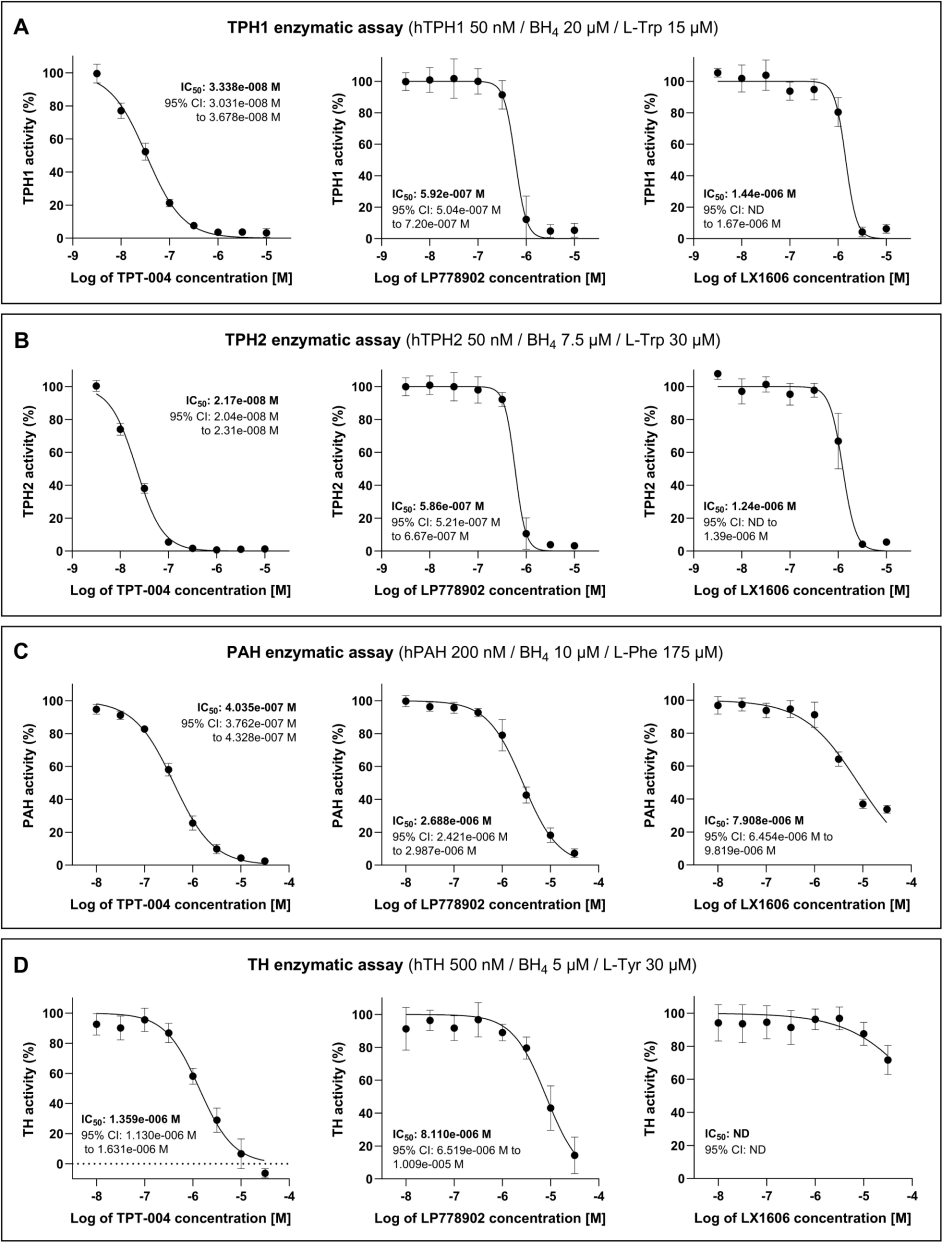
Potency and selectivity profiling of TPT-004, LP778902, and LX1606 in AAAH assays. Enzymatic assays for (A) TPH1, (B) TPH2, (C) PAH, and (D) TH. Data points represent mean enzymatic activity calculated from at least four independent experiments ± SD (detailed information in Supplementary Tables S2–S5). A nonlinear regression model (log­(inhibitor) vs normalized response – variable slope) was fitted to the data, and the IC_50_ value was determined including the 95% confidence interval (CI). ND = not defined.

Next, TPT-004, LP778902, and LX1606 were tested in the concentration range from 10 nM to 30 μM in the PAH and TH assays. In the PAH assay (Figure 1C), TPT-004 and LP778902 showed dose-dependent inhibition of the enzyme, whereas LX1606, despite displaying a similar trend, did not reduce PAH activity below 30%, even at the highest tested concentration. The relative enzymatic activity of PAH after treatment with different concentrations of TPT-004, LP778902, and LX1606 is shown in Table S4. The IC_50_ of TPT-004 in the PAH assay was determined to be 4.035 × 10^–07^ M, while the PAH IC_50_ of LP778902 was 2.688 × 10^–06^ M. The PAH IC_50_ of LX1606 was determined to be 7.908 × 10^–06^ M.

In the TH assay (Figure 1D), TPT-004 and LP778902 exhibited a dose-dependent inhibition of TH. The relative enzymatic activity of TH after treatment with different concentrations of the compounds is shown in Table S5. The TH IC_50_ of TPT-004 was 1.359 × 10^–06^ M, whereas the IC_50_ of LP778902 was 8.110 × 10^–06^ M. In contrast, LX1606 showed only a minor inhibitory effect at the two highest concentrations (10 and 30 μM), with TH activity not reduced by more than 30% compared to the positive control. Consequently, determining the IC_50_ was not feasible.

Based on the IC_50_ values determined in the AAAH enzymatic assays, we calculated the selectivity factor between TPH1 and other AAAH family members for each compound (Table 1). Our data show that LP778902 is 13.7 times more selective toward TPH1 than TH and 4.54 times more selective toward TPH1 than PAH, but it lacks specificity between TPH1 and TPH2 (0.99×). LX1606 shows a similar selectivity between TPH2/TPH1 and PAH/TPH1 as its active drug form LP778902. As LX1606 did not inhibit TH activity (>50%) in the tested concentration range and the IC_50_ value could not be calculated, the corresponding TH/TPH1 selectivity factor has not been defined. TPT-004, on the other hand, has an almost 18× higher inhibitory potency for the TPH1 enzyme compared to the active form of Telotristat (LP778902) (33.38 vs 592 nM, Figure 1) and also displays a better TH/TPH1 (40.71 vs 13.70x) and PAH/TPH1 selectivity factor (12.09× vs 4.54×). This indicates a potentially broader therapeutic window for TPT-004 to reduce peripheral serotonin levels with a sufficient safety margin to avoid potential adverse effects from PAH or TH inhibition.

**Table 1:**
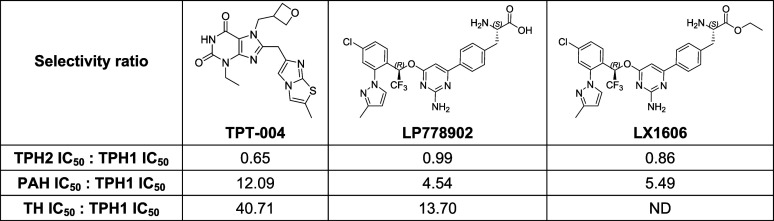
TPH1 Selectivity Factor Calculated Based on the Determined IC_50_ values for TPT-004, LP778902, and LX1606 in the AAAH Enzymatic Assays^a^

a ND = not defined

Comparable to that of LP778902, TPT-004 exhibits similar IC_50_ values for both TPH1 and TPH2. Given its inhibitory effect on the TPH2 isoform, preventing TPT-004 from penetrating the blood-brain barrier is advisable. Previously published pharmacokinetic data indicate exceptionally low blood-brain barrier penetration by TPT-004 in two rodent species.^22^ Moreover, prior pharmacodynamic studies with TPT-004 in mice did not show an altered balance of brain serotonin turnover in the dose–range of 1.5 mg/kg bis in die (BID, two times daily) to 100 mg/kg BID. Additionally, no neuropsychiatric effects were observed following a battery of behavioral tests in mice after 14 days of repeated oral treatment with TPT-004 at doses of 10 or 50 mg/kg BID.

### Structural Insights into Inhibitor Selectivity

Our previous analyses of TPH1 complex crystal structures revealed a tripartite binding mode of the lead inhibitor TPT-004, spanning the binding sites of the cosubstrate pterin, the substrate tryptophan, and chelating the catalytic iron ion.^22^ TPT-004 exploits two hydrophobic active site subpockets for binding: Subpocket 1, naturally occupied by the polar, aliphatic extension of the cosubstrate pterin, is flanked by Phe241, Leu242, Ala309, and Tyr312 and is involved in R^1^ chain binding (the ethyl group in TPT-004). Subpocket 2 is flanked by Val232, Tyr235, Thr253, Tyr255, and Pro268 and is involved in R^2^ chain binding (the oxetane ring in TPT-004). The R^3^ portion of TPT-004 (the xanthine–imidazothiazole group) localizes to the substrate-binding site (Figure 2A).^22^ Telotristat, on the other hand, primarily targets the substrate-binding site of TPH1. Importantly, Telotristat binding does not address the two active site subpockets, 1 and 2, as compared to TPT-004 (Figure 2B).^21^

**Figure 2:**
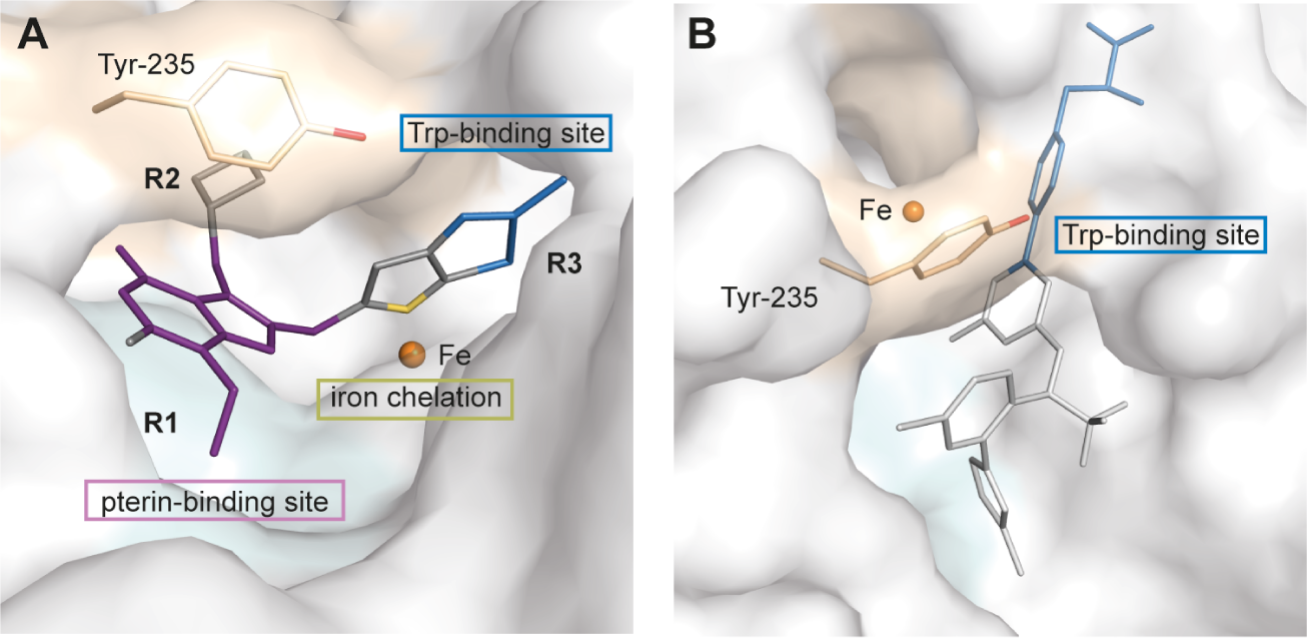
Binding mode of lead TPH1 inhibitors. Inhibitors are shown as stick models. The part of the inhibitor that binds into the binding site of the cosubstrate pterin or substrate tryptophan are colored in purple or blue, respectively, and the iron-chelating moiety is colored in yellow. The TPH1 surface is shown in gray, and the iron ion is shown as an orange sphere. The two hydrophobic subpockets are displayed as colored surface representations (subpocket 1 in blue, subpocket 2 in orange). (A) TPT-004 exploits the cosubstrate and the substrate binding site (R^3^), and chelates the iron ion (PDB ID code: 8CJL.^22^ The R^1^ and R^2^ moieties bind into the active site subpockets 1 and 2, respectively. (B) Telotristat (LP778902) primarily targets the substrate-binding site (PDB ID code: 7ZIK).^21^

In order to understand and rationalize the experimental results regarding inhibitor selectivity, we performed a systematic analysis of the published TPH1, PAH, TH, and the herein reported TPH2 crystal structures (Table S9) and mapped residues lining the TPH1/2 active site onto the sequence alignment of the four human AAAH enzymes (Figure 3). Our structural analysis confirms that the key binding interactions observed in TPH1 are also present in TPH2, demonstrating that their active site residues are conserved both in amino acid sequence (Figure 3) and spatial positioning within the active site (Figure S1). However, differences in the core active sites of PAH and TH help explain the superior selectivity ratios of TPT-004 for TPHs within the AAAH enzyme family (Table 1). Following this, we will present the results by examining key regions of the active site. The discussion is structured into four sections: (i) amino acid residues located at the rim of the active site; (ii) residues lining the substrate or cosubstrate-binding site; (iii) subpocket 1, which accommodates specific inhibitor moieties and plays a role in selectivity; and (iv) subpocket 2, which provides additional binding interactions that differentiate inhibitor affinities among the enzymes.

**Figure 3:**
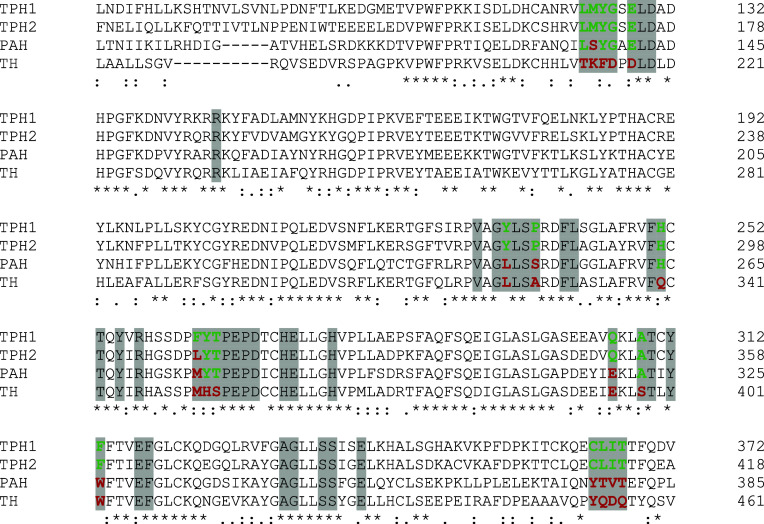
Amino acid sequence alignment of human TPH1, TPH2, PAH, and TH. Residues lining the active site, defined by their distance of 5 Å around the complexed ligands (BH_4_, inhibitor, substrate, or Fe) in the reported TPH1 crystal structures (PDB ID codes: 1MLW,^29^ 3HF6,^30^ 3HF8 and 3HFB,^31^ 5J6D,^32^ 5L01 and 5TPG,^33^ 7ZIF-7ZIK,^21^ and 8CJI-8CJO),^22^ are shaded in gray with nonconserved AAAH active site residues highlighted in color (green: TPH1-like, red: different from TPH1). The overall alignment scores for full-length proteins in percentage are 70.50 for TPH1 versus TPH2, 55.30 for TPH1 versus PAH, 49.65 for TPH1 versus TH. The multiple sequence alignment was done using Clustal Omega.^34^

Amino acid residues are located at the rim of the active site: the sequence alignment reveals Phe263 (TPH2:Leu309) as the only differing residue between TPH1/2 (Figure 3) located at the entrance of the active site. Alongside Thr176, Val177, Arg257, Phe263, Tyr264, Thr265, and Ser336-Ser339, it forms a solvent-exposed channel that extends into the substrate-binding site. Phe263 interacts with inhibitors via main-chain interactions, not affecting the selectivity. Structural analysis shows decreasing hydrophobicity of the loop TPH1:Phe263-Thr265 (TPH2:Leu309-Thr311, PAH:Met276-Thr278, and TH:Met352-Ser354) across the AAAH family (Figure 3, TPH1 > TPH2 > PAH > TH), potentially regulating access to and shielding of the active site (Figure 4A). Additional variations include the loop TPH1:Leu123-Glu128 (TPH2:Leu169-Glu174, PAH:Leu136-Glu141, and TH:Thr212-Asp217) that is conserved in TPH1/2, partially conserved in PAH except for the Ser137 residue, but different in TH (Figure 3). The loop is not resolved in the TPH1 structures in complex with TPT-004 and Telotristat but may as well modulate access to and shield the active site from solvent exposure (Figure 4A). Another residue, TPH1-Gln306 (Gln352/Glu319/Glu395) is conserved in TPH1/2 but replaced by a glutamate in PAH and TH (Figure 3), introducing a negative charge that could influence the electrostatic environment (Figure 4A). While rim residues impact the accessibility, primary specificity is mainly influenced by residues deep in the active site, where key binding interactions occur.

**Figure 4:**
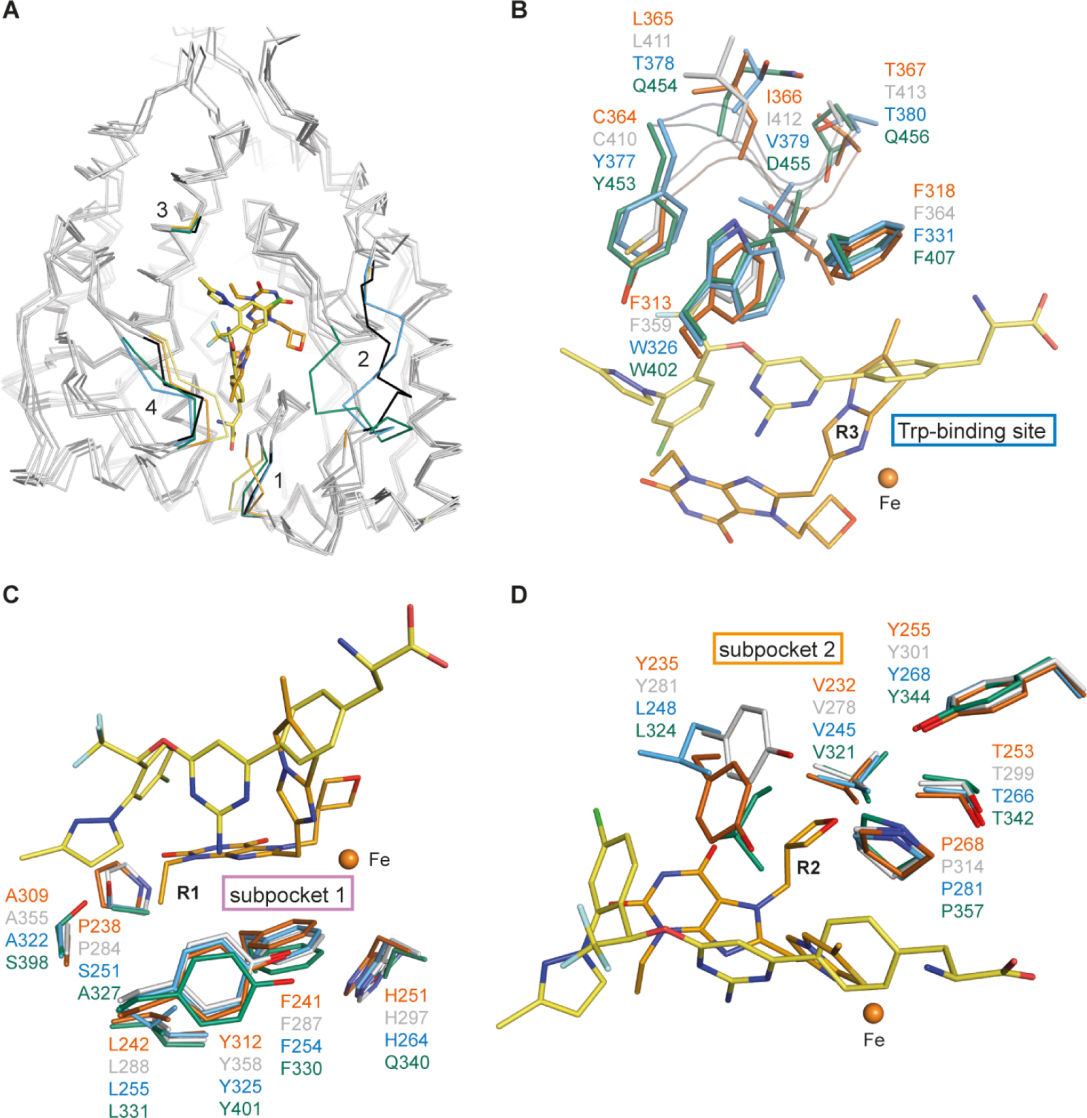
Superimposition of various human AAAH crystal structures. The crystal structures of human TPH1 bound to TPT-004 (PDB ID code: 8CJL;^22^ C: orange, O: red, N: blue) or Telotristat (PDB ID code: 7ZIK;^21^ C: yellow, O: red, N: blue, Cl: green, and F: light blue), human TPH2 (PDB ID code: 4 V06; C: black or gray, O: red, and N: blue), human PAH (PDB ID code: 1PAH;^35^ C: sky blue, O: red, and N: blue), and human TH (PDB ID code: 2XSN; C: green, O: red, and N: blue) are superimposed. Inhibitors and amino acid residues with a possible impact on selectivity are shown as stick models, and the iron ion as orange sphere. The color of the amino acid residue labels corresponds to the color of the carbon atoms of each enzyme (TPH1, orange; TPH2, gray; PAH, sky blue; and TH, green). (A) The superimposed human AAAH crystal structures are shown as gray ribbon models. Highlighted are distinct loop regions and amino acids: 1, loop Phe263-Thr265; 2, loop Leu123-Glu128 (not resolved in TPH1 structures); 3, Gln306; 4, loop Cys364-Thr367; numbering shown only for TPH1, refer to the text for others. Amino acids potentially involved in specificity: (B) in the substrate-binding pocket, (C) in subpocket 1, and (D) in subpocket 2.

Amino acid residues lining the substrate or cosubstrate-binding site: The amino acid side chain of human TPH1:Phe313 (Phe359/Trp326/Trp402 in human TPH2, PAH, and TH, respectively; the same order of numbering applies for the discussion below; Figure 3) is positioned near the binding pocket of the respective substrate, where, together with TPH1:Phe318 (Phe364/Phe331/Phe407), it forms hydrophobic π–π interactions (Figure 4B). In the complex structure with Telotristat, Phe313 interacts with the amino-pyrimidine ring of the inhibitor, but it does not reach the heterocyclic imidazothiazole ring in the TPT-004 complex structure (Figure 4B). Because of the similar aromatic nature of Phe (in TPH1/2) and Trp (in PAH/TH), their impact on the selectivity can be considered negligible. The peripheral loop TPH1:Cys364-Thr367 (TPH2:Cys410-Thr413, PAH:Tyr377-Thr380, TH:Tyr453-Gln456), with proximity to the substrate-binding pocket (Figure 4A), is conserved in TPH1/2 but completely different in PAH and TH (Figure 3). The side chain of TPH1:Ile366 (Ile412, Val379, and Asp455) makes hydrophobic interactions with the R^3^ part of TPT-004 and the trifluoromethoxyphenyl-aminopyrimidine moiety of Telotristat (Figure 4B). The substitution by Val379 in PAH weakens these interactions, and the substitution by Asp455 in TH (which additionally forms a salt bridge with Lys213 of the opposing active site loop) disrupts these hydrophobic interactions. Moreover, the trifluoromethyl group of Telotristat is located near TPH1:Cys364 (Cys410, Tyr377, Tyr453). The bulkier side chain of the positionally conserved tyrosine residue in PAH and TH could interfere with Telotristat but not with TPT-004 binding (Figure 4B). The amino acid residue TPH1:His251 (His297/His264/Gln340) is located near the cosubstrate-binding site but is not involved in direct inhibitor interactions and, thus, is unlikely to contribute to selectivity.

Amino acid residues in subpocket 1 of the TPH1/2 active site: The residue variations at TPH1:Pro238 (Pro284/Ser251/Ala327) and TPH1:Ala309 (Ala355/Ala322/Ser398) (Figure 3) further contribute to selectivity. Both nonpolar side chains are part of a hydrophobic subpocket in the active site, accommodating the ethyl group of TPT-004 attached to the xanthine ring (Figure 4C). The corresponding polar and hydrophilic serine residues in PAH and TH decrease the hydrophobic character of the subpocket, likely creating less favorable interactions for inhibitor binding. Telotristat, on the other hand, does not bind into the above-mentioned hydrophobic subpocket 1 (Figure 4C).

Amino acid residues in subpocket 2 of the TPH1/2 active site: The amino acid side chain of human TPH1:Tyr235 (Tyr281/Leu248/Leu324) forms strong π–π and π-alkyl interactions with the xanthine and oxetane rings of TPT-004, respectively, while the possible hydrophobic interactions of the positionally conserved leucine residues in PAH and TH are much weaker (Figures 3 and 4D). In the case of the Telotristat complex structure, TPH1:Tyr235 forms π–π interactions with the amino-pyrimidine ring as well as with the chloro-phenyl ring, compared to the much weaker alkyl-π interactions in the case of the leucine residues in PAH and TH (Figures 3 and 4D), highlighting the importance of Tyr235 for TPH1 affinity and selectivity. Future studies will focus on mutagenesis experiments, swapping Tyr for Leu in TPH and Leu for Tyr in PAH and TH to better understand the extent to which these amino acids contribute to enzyme selectivity.

Previous structural studies have demonstrated that the N-terminal domains in AAAH enzymes regulate catalytic activity through allosteric inhibition, conformational control, and modulation of access to the active site.^36–40^ For example, the N-terminal regulatory domains (RDs) of TPH1, TPH2, and TH are able to form dimers, while TPH2 and PAH display substrate-binding sites in their RDs.^13,39,41^ Cryo-EM structures of full-length TPH2, PAH, and TH enzymes reveal flexible RD interactions with the catalytic domain, controlling active site access depending on modifications such as phosphorylation or ligand binding. The structural dynamics of the regulatory N-terminal domains suggest that the RDs may play an additional role in influencing enzyme specificity.

### Broad Selectivity Profiling *In Vitro*

Next, we evaluated TPT-004 using the Eurofins Diversity Panel, which enables *in vitro* profiling of drug candidates against a wide range of targets, including many relevant to safety. The results of screening TPT-004 at 10 μM against 97 targets are shown in Figures 5 and 6. Hits showing more than 50% inhibition or stimulation are considered to represent significant effects of the test compounds. Out of the 97 targets, a significant effect was observed only for the rat homolog of TH, with 87.8% activity inhibition at 10 μM of TPT-004. This finding was anticipated and is consistent with our results on the partial inhibitory activity of human TH at the higher tested concentrations of TPT-004 (Figure 1). The absence of other significant screening hits indicates that TPT-004 has excellent target selectivity. The European Medicines Agency’s (EMA) assessment of Xermelo® [EMA/508026/2017] reveals that Telotristat ethyl (the prodrug form LX1606) and LP778902 (its active metabolite) underwent testing for interactions with 75 human receptors, enzymes, and ion channels.^42^ The prodrug LX1606 inhibited over 50% of the activity for 16 of 75 receptors, indicating a high *in vitro* interaction potential. LP778902 showed weaker interaction potential with these receptors; however, a >50% inhibition was reported at 10 μM for the A3, 5-HT1B, and 5-HT2A receptors.

**Figure 5:**
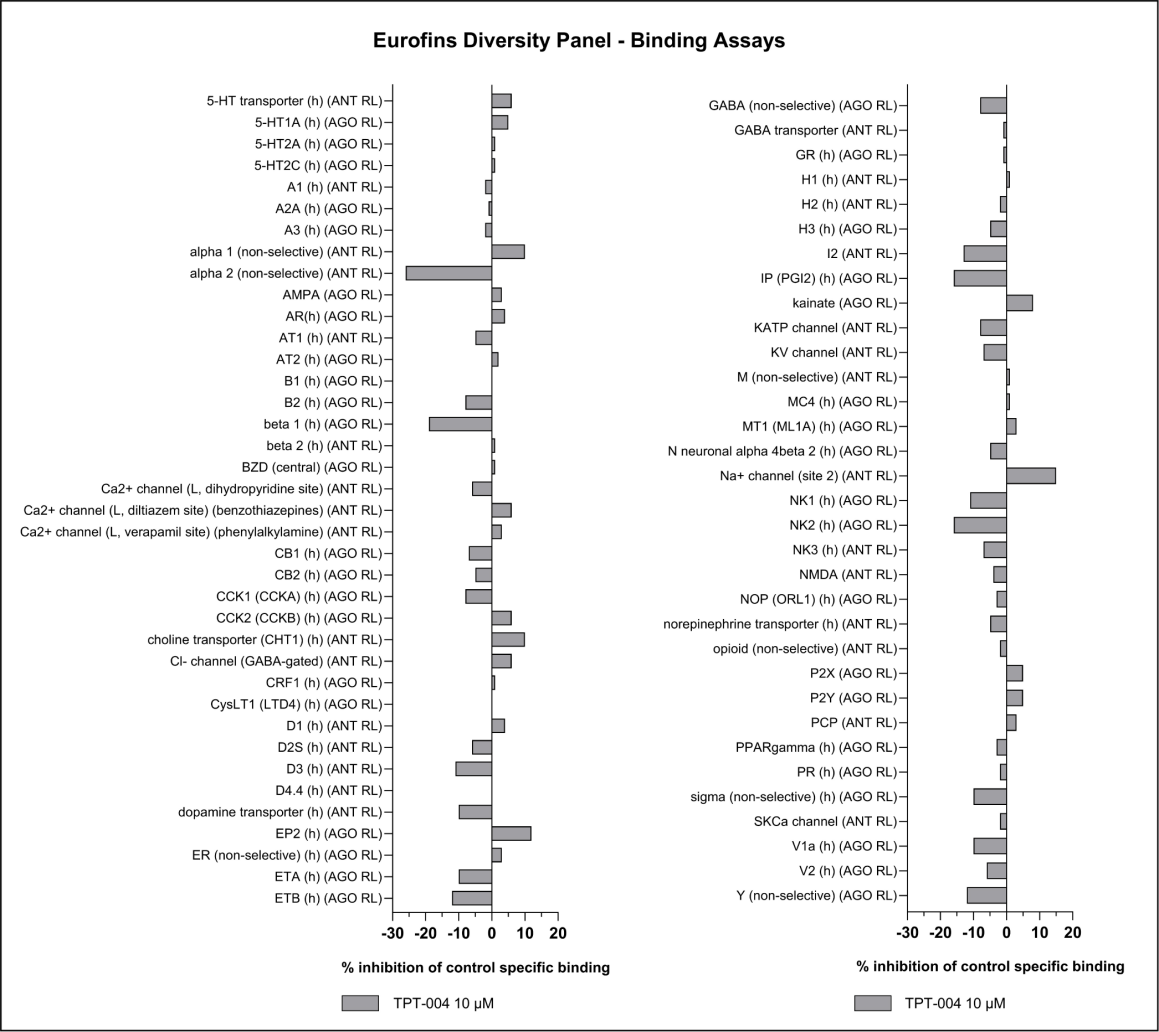
Broad selectivity screening applying binding assays with TPT-004. Displayed are the results from the Eurofins Diversity Panel performed at 10 μM. ANT RL = antagonist radioligand; AGO RL = agonist radioligand.

**Figure 6:**
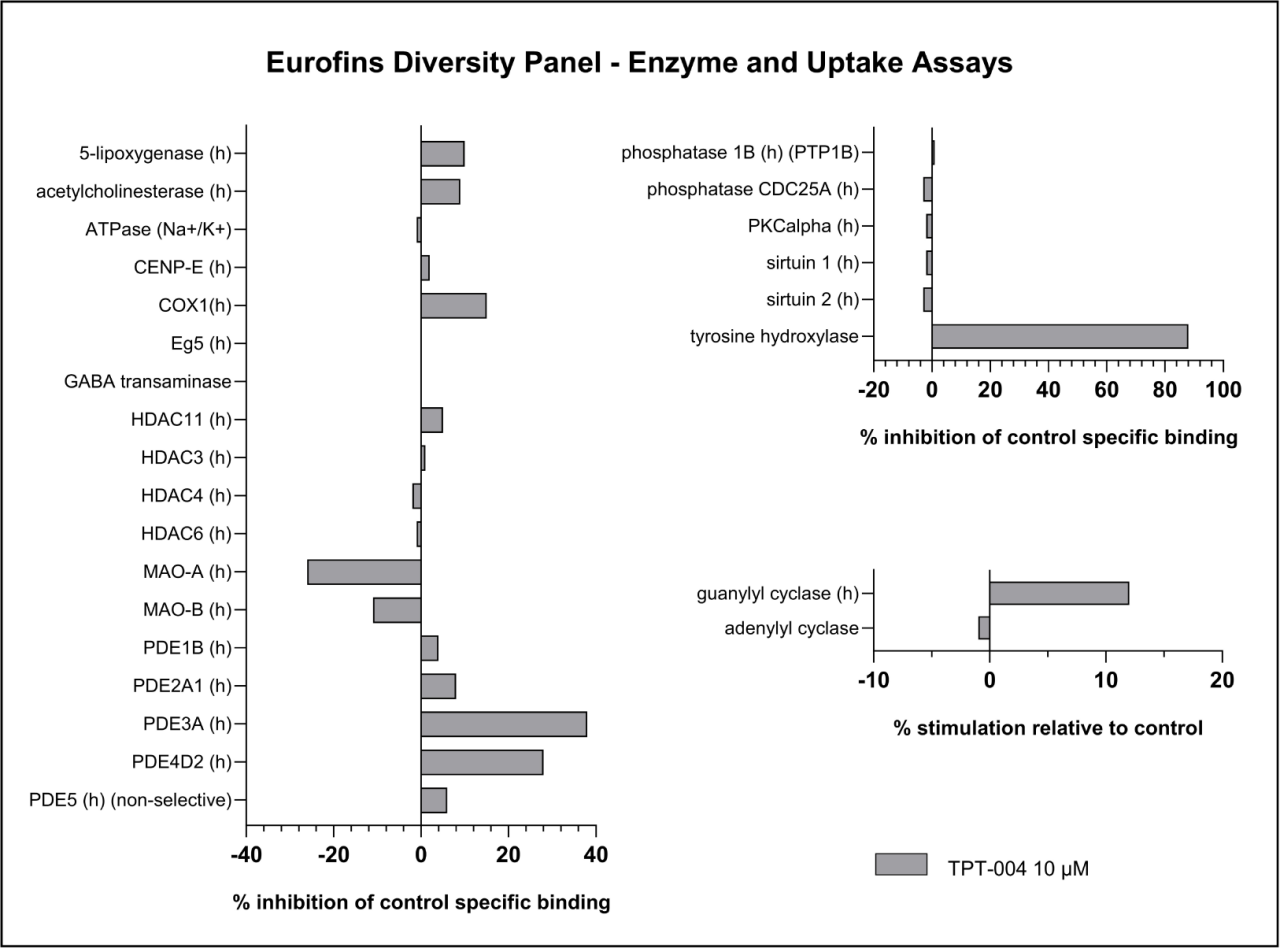
Broad selectivity screening applying enzyme and uptake assays of TPT-004. Displayed are the results from the Eurofins Diversity Panel performed at 10 μM.

### Dose–Range Finding (DRF) Study in Rats

In the final step, we proceeded with the toxicity evaluation after repeated dosing of TPT-004 in Sprague–Dawley rats. TPT-004 was administered by oral gavage (PO) twice daily (BID) with a 12-h interval to the treatment groups (3 male and 3 female Sprague–Dawley rats each) for 8 days (15 successive applications). Rats were dosed with 10, 45, or 200 mg/kg BID, resulting in total daily doses of 20, 90, and 400 mg/kg, respectively. A control group, consisting of the same number and sex distribution of animals, was administered the vehicle (0.5% CMC-Na, sodium carboxymethyl cellulose) at the same dosing frequency.

All of the animals survived the scheduled study period. None of the rats exhibited clinical signs during the experiment, and their body weight increased normally during the course of the study (Figure 7A). The food consumption by the TPT-004-treated rats was in the same range as that of the control group (Figure 7B). The repeated oral application of TPT-004 at 10, 45, or 200 mg/kg BID did not alter the hematology parameters (Figure 8A and Table S6). The values in the treated groups were similar to those in the control group. The analysis of red cell distribution width (RDW) showed a slight yet statistically relevant elevation in the animals treated with TPT-004 at 200 mg/kg BID (19.3 ± 1.6 fl. vs 16.4 ± 0.8 fl. in the vehicle group; *p* = 0.0109). The number of platelets (PLT) is the only parameter where larger differences were observed; however, these differences were not significant level. The clinical chemistry parameters also showed no relevant difference between the vehicle and treatment groups (Figure 8B and Table S7).

**Figure 7:**
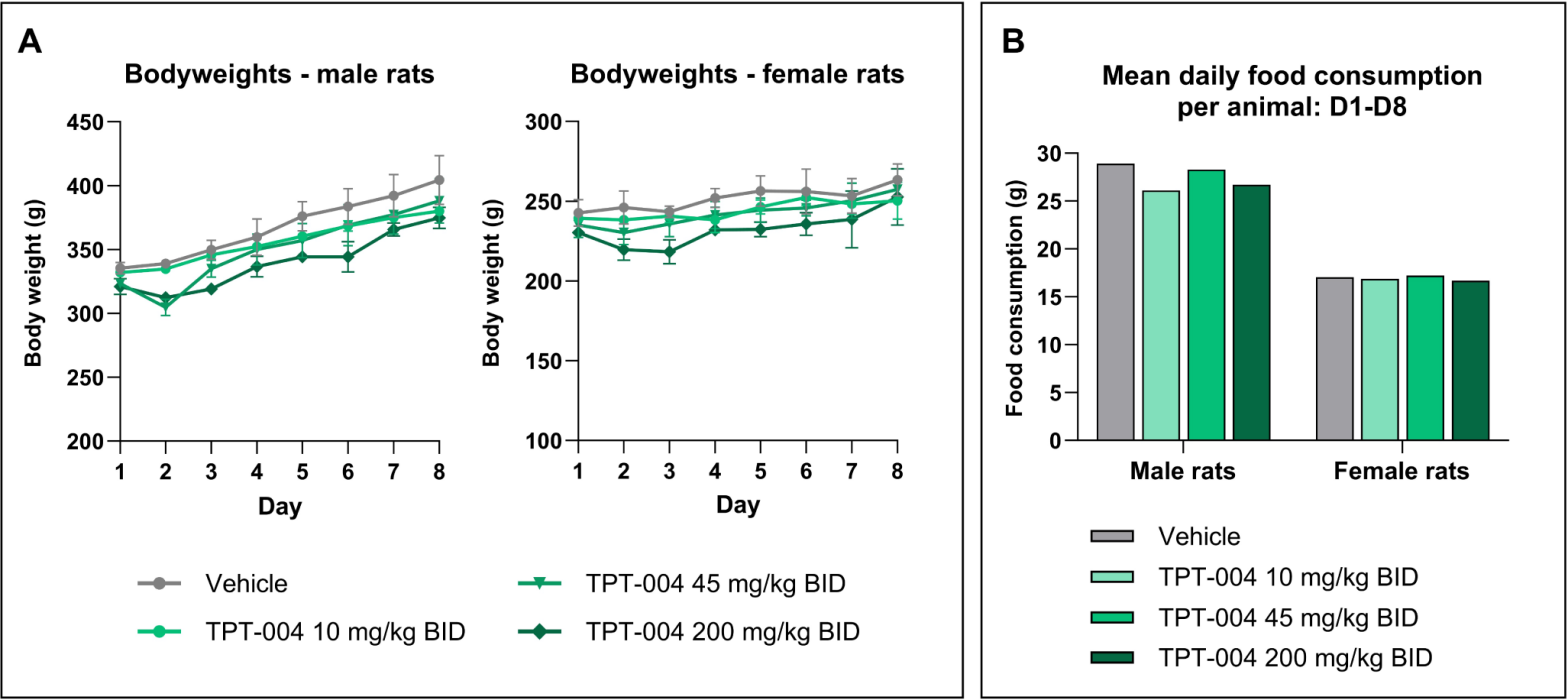
Results of the in-life phase of the dose–range finding study in Sprague–Dawley rats after subchronic TPT-004 treatment. The animals were dosed with TPT-004 (10, 45, and 200 mg/kg BID) for 8 days (15 successive applications). (A) Mean bodyweight between D1 and D8 in male (*n* = 3) and female (*n* = 3) rats per group. (B) Average daily food intake between D1 and D8 male (*n* = 3) and female (*n* = 3) rats per group.

**Figure 8:**
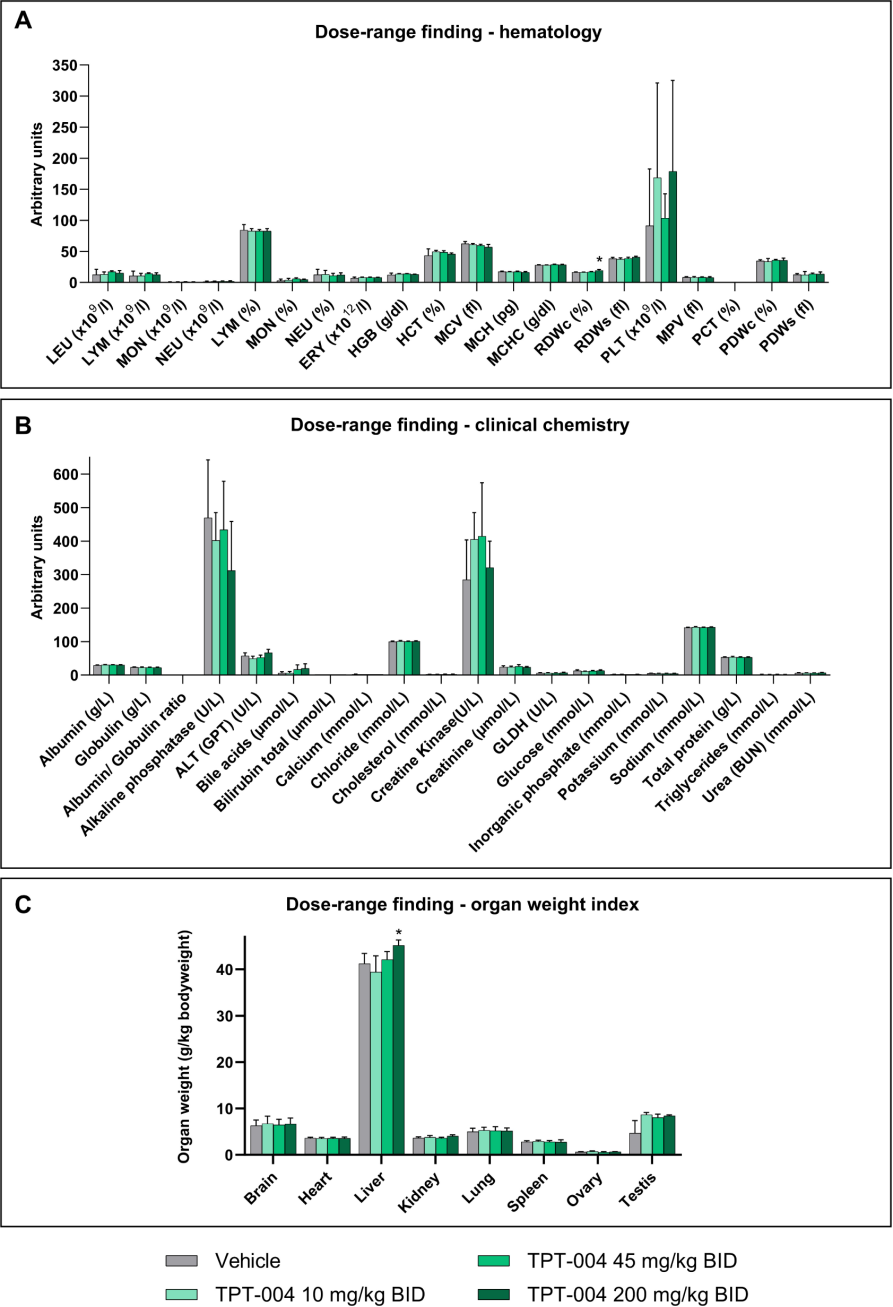
Results of the bioanalytical phase of the dose–range finding study in Sprague–Dawley rats after subchronic TPT-004 treatment. The animals (*n* = 3 males and *n* = 3 females per group) were dosed with TPT-004 (10, 45, and 200 mg/kg BID) for 8 days (15 successive applications). (A) Mean (±SD) hematology parameters. (B) Mean (±SD) clinical chemistry parameters. (C) Mean (±SD) organ weight index at necropsy. Mixed effects analysis with Dunnett’s multiple comparison test; *p < 0.0332.

No test-item-related gross lesions were recorded during necropsy. No significant difference was observed in the weight of the organs between the vehicle and treatment groups, except for slightly elevated liver weights in the rats treated with TPT-004 at 200 mg/kg BID (45.2 ± 1.2 g/kg BW vs 41.2 ± 2.2 g/kg BW in the vehicle group; *p* = 0.0128) (Figure 8C and Table S8). Based on the histopathological assessment, there were no test-related findings in the heart, spleen, kidney, lung, brain, femur, testis, or ovary of the animals treated with TPT-004 at 45 and 200 mg/kg BID. However, in the liver, there was a minimal increase in the size of hepatocytes around the centrilobular areas only at doses ≥45 mg/kg BID, characterized by larger nuclei, indicative of multifocal, minimal centrilobular hypertrophy (Figure S2). This hepatocellular centrilobular hypertrophy was considered to be induced by the test item and associated with an adaptive response to an increase in hepatic metabolism.^43^ This type of change would be expected to spontaneously resolve within a few days after cessation of treatment. Overall, the presented data suggest a promising safety margin for TPT-004, especially considering the low doses needed to achieve the therapeutic effects in mouse colorectal carcinoma (50 mg/kg BID)^22^ and the Sugen–Hypoxia rat model of pulmonary arterial hypertension (10 mg/kg BID).^44^

In addition to the toxicity assessment, we evaluated the plasma exposure levels of TPT-004 after a single dose and at the end of the in-life phase (after receiving 15 doses). On day 1, 30 min after the first application, the mean plasma concentration of TPT-004 was calculated to be 902 ± 247 ng/mL for the 10 mg/kg dose, 4722 ± 2415 ng/mL for the 45 mg/kg dose, and 18235 ± 6614 ng/mL for the 200 mg/kg dose. On day 8, 30 min after the 15th and last application, the mean plasma concentration of TPT-004 was calculated to be 877 ± 210 ng/mL for the 10 mg/kg BID dose, 5038 ± 1786 ng/mL for the 45 mg/kg BID dose, and 14358 ± 5601 ng/mL for the 200 mg/kg BID dose (Figure 9A). TPT-004 was not detected in the vehicle group. These data demonstrate the absence of an accumulation effect, with dose linearity exhibiting correlations of 0.9987 on day 1 and 0.9826 on day 8 (Figure 9B).

**Figure 9:**
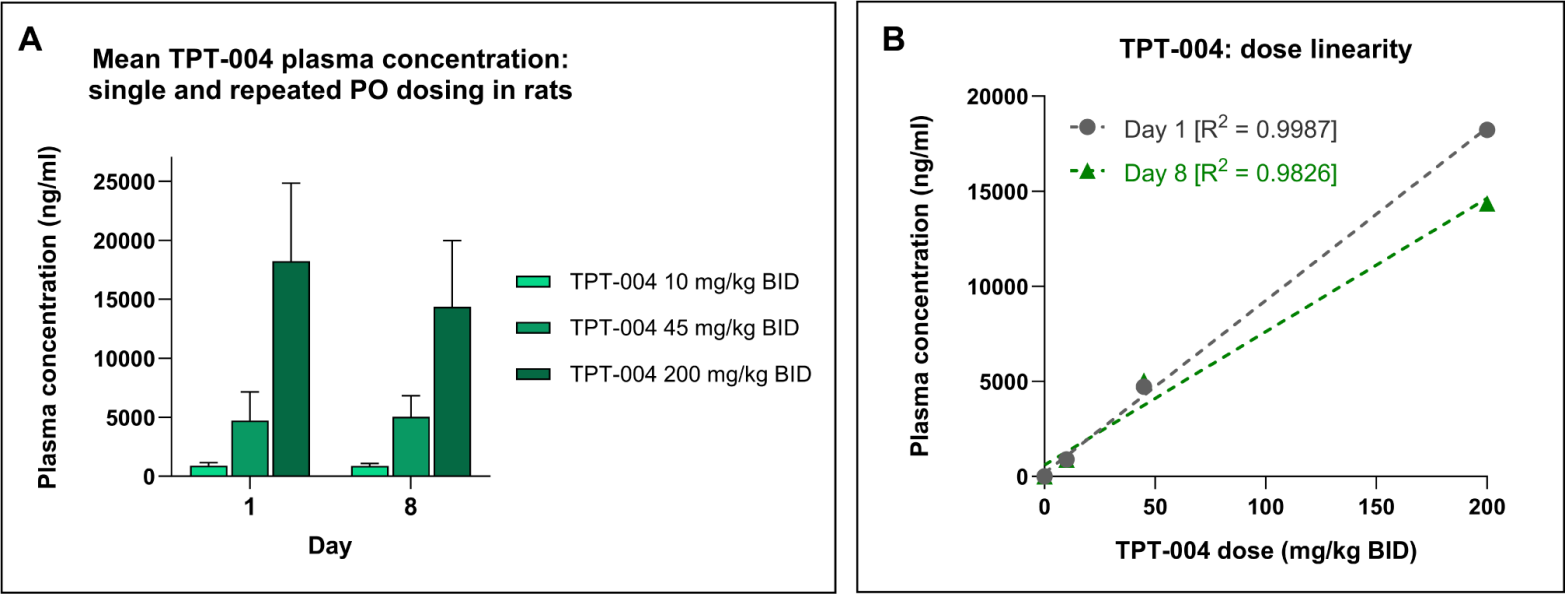
Toxicokinetic evaluation of TPT-004 in Sprague–Dawley rats. The animals (*n* = 3 males and *n* = 3 females per group) were dosed with TPT-004 (10, 45, and 200 mg/kg BID) for 8 days (15 successive applications). (A) The mean plasma concentration of TPT-004 after the first and last oral dose. The exposure levels (ng/mL or ng/g) of TPT-004 are represented as mean ± SD. (B) Dose-linearity of TPT-004 in plasma after the first and last oral dose.

## Conclusions

In conclusion, this comprehensive evaluation underscores the potential of TPT-004 as a highly selective and effective inhibitor of tryptophan hydroxylases (TPHs), with promising therapeutic prospects for managing serotonin-related disorders. Through the development of optimized, high-throughput fluorescence assays, we demonstrated that TPT-004 exhibits exceptional potency and selectivity for TPH1 compared with other members of the AAAH family. Specifically, TPT-004 showed a significant improvement in TPH1 inhibition, with an IC_50_ value 18 times lower than that of the clinically approved TPH inhibitor Telotristat (LP778902). Moreover, TPT-004 achieved superior selectivity ratios, indicating a broader therapeutic window with a reduced risk of off-target effects on PAH and TH enzymes. The sequence alignment of the four AAAH enzymes, along with the solved cocrystal structures of TPH1 and TPH2 bound to different inhibitors, reveals that selectivity is primarily driven by variations in the deep active site, particularly within subpockets 1 and 2 of TPH, where key inhibitor interactions occur.

The comprehensive selectivity profiling of TPT-004 against 97 targets further confirms its specificity, revealing minimal significant interactions beyond the anticipated partial inhibition of TH. This high selectivity is crucial for minimizing adverse effects and improving therapeutic outcomes. Additionally, the dose range-finding (DRF) study in rats demonstrated an acceptable safety profile, with no severe clinical signs or significant toxicological findings, even at the highest tested dose of 400 mg/kg. The observed hepatocellular changes were minimal and likely adaptive, suggesting that they would resolve post-treatment.

Collectively, these results indicate that TPT-004 has a promising profile for addressing conditions associated with excessive peripheral serotonin synthesis. Its high potency, selective inhibition of TPHs, and favorable safety margin position it as a lead candidate for further clinical development. Future studies will be essential to confirm these findings in human trials and fully explore its potential as a therapeutic agent for serotonin-related diseases.

## Materials and Methods

### Recombinant Protein Production

Full-length human TPH1 and TPH2 (UniProt entries P17752 and Q8IWU9) were produced as N-terminally MBP-tagged proteins, as published earlier.^21,45^ The construct comprising the isolated catalytically active and oligomerization domain of human TPH2 (aa 148–490) was produced as a His_7_-tagged protein (subcloned via BamHI/NotI into the pQLinkH expression vector)^46^ using *E. coli* Rosetta (DE3) cells (Novagen). TB media (Terrific Broth) was supplemented with 100 μg/mL ampicillin and 34 μg/mL chloramphenicol. The cultures were grown at 37 °C until the OD_600_ reached about 2.5, using a LEX ultrahigh-throughput benchtop bioreactor (Epiphyte3). Gene expression was induced by the addition of 0.5 mM IPTG at 17 °C. After induction, cultures were grown overnight at 17 °C. Cells were harvested by centrifugation, and the pellets were stored at −80 °C. For purification, cells were resuspended in lysis buffer (50 mM Hepes-NaOH pH 7.5, 0.5 M NaCl, 5% v/v glycerol) supplemented with 2.5 U/mL benzonase (Merck KGaA), 1 mM DTT, 0.1 mM PMSF, and cOmplete (EDTA-free protease inhibitor cocktail, Roche), and lysed by sonication (SONOPULS HD 2200, Bandelin). After centrifugation, the supernatant was supplemented with 10 mM imidazole pH 8.0 and applied onto a 5 mL HisTrap FF crude column (Cytiva) pre-equilibrated with lysis buffer. After washing with 5 column volumes of the same buffer, first supplemented with 20 mM and then 50 mM imidazole, the protein was eluted using lysis buffer containing 250 mM imidazole. The eluate was supplemented with 2 mM DTT and applied onto a Superdex 200 prep grade column (XK 26 × 600, Cytiva) pre-equilibrated with lysis buffer. The protein was further purified via anion exchange chromatography on a 5 mL Source 30Q column (Cytiva) equilibrated with 50 mM HEPES-NaOH pH 7.5, 5% v/v glycerol, 50 mM NaCl, and 2 mM DTT. Bound proteins were eluted using a linear gradient from 50 mM NaCl to 1 M NaCl, and the purified protein was concentrated to about 4 mg/mL, flash-frozen with liquid nitrogen, and stored at −80 °C until usage.

The pMAL-c5E expression vectors for full-length human PAH (UniProt entry P00439) and human TH (UniProt entry P07101) were produced by gene synthesis (GenScript). The resulting TEV-cleavable, N-terminally MBP-tagged proteins were produced using *E. coli* T7 Express cells (NEB) cotransformed with the pRARE2 plasmid (Novagen). TB medium was supplemented with 1% glucose (v/v), 100 μg/mL ampicillin, and 34 μg/mL chloramphenicol. The cultures were grown at 37 °C until the OD_600_ reached approximately 2. Gene expression was induced by the addition of 0.5 mM IPTG at 17 °C. After induction, cultures were grown overnight at 17 °C. Cells were harvested by centrifugation. For purification of MBP-tagged full-length PAH and TH, cells were resuspended in lysis buffer (50 mM HEPES, pH 6.8, and 100 mM NaCl), supplemented with 0.5–1 mM DTT, 1 mM PMSF, 2.5–8.5 U/mL benzonase, and 16.500 U/mL lysozyme, and lysed by either two freeze–thaw cycles (PAH) or by sonication (TH, SONOPULS HD 2200, Bandelin Electronic GmbH & Co. KG). After centrifugation, the supernatant was applied onto a 2 × 5 mL MBPTrap HP column (Cytiva) pre-equilibrated with lysis buffer. After washing the column with 10–12 CV of lysis buffer, the protein was eluted in the same buffer containing 10 mM maltose. The eluate was supplemented with 2 mM DTT, and applied onto a Superdex 200 prep grade column (XK 26 × 60, Cytiva) pre-equilibrated with 50 mM HEPES, pH 6.8, and 300 mM NaCl. After the addition of 1 mM DTT, purified proteins were concentrated to 2–3 mg/mL, flash-frozen with liquid nitrogen, and stored at −80 °C until use.

### Protein Crystallization and Structure Determination

For complex crystallization, the TPH2 protein was diluted to 1 mg/mL and mixed with 0.5–1.0 mM inhibitor, followed by coconcentration to 11 mg/mL. The protein–inhibitor complex was crystallized using the sitting-drop vapor-diffusion method at 20 °C by mixing equal volumes (200 nL) of the protein–inhibitor complex and reservoir solution (AG-01-128, inhibitor **29** in^21^ 20% w/v PEG 3350, 200 mM sodium acetate, 0.1 M BisTrisPropane, pH 7.0, 10% v/v ethylene glycol; KM-06-098, inhibitor **16** in^22^ 17.5% w/v PEG 3350, 200 mM sodium acetate, 0.1 M BisTrisPropane, pH 7.0, 10% v/v ethylene glycol). Before flash-freezing in liquid nitrogen, the crystal was transferred into a cryoprotectant consisting of the reservoir solution supplemented with 15–25% (v/v) ethylene glycol and 13–22% (v/v) glycerol.

Diffraction data were collected at 100 K at beamline BL14.1, operated by the Helmholtz-Zentrum Berlin (HZB) at the BESSY II electron storage ring (Berlin-Adlershof, Germany),^47^ using a wavelength of 0.9184 Å. Data were processed with the program Xdsapp.^48^ The structure of the catalytic and oligomerization domain of human TPH2 was solved by molecular replacement using the program Phaser^49^ and the known human TPH2 crystal structure with PDB ID code 4v06 as the search model. The structure was refined using PHENIX^50^ and the graphics program Coot was used for model building and visualization.^51^ The Prodrg server^52^ was used to generate topology files for the ligands. Data collection and refinement statistics are reported in Table S9. Figures were created with Pymol.^53^

### TPH1 Activity Assay

The TPH1 enzymatic activity assay was performed using a 96-well plate format (Greiner). The TPH1 reaction mixture (total volume of 200 μL) contained 50 mM MES buffer (pH 7.0) (Sigma-Aldrich), 50 μM FAS (Sigma-Aldrich), 20 μM BH_4_ (Cayman Chemicals), 0.05 mg/mL catalase (Sigma-Aldrich), 5 mM DTT (Sigma-Aldrich), and 50 nM human recombinant TPH1 enzyme. To initiate the reaction, l-Trp (Sigma-Aldrich) was added to a final concentration of 15 μM. The assay contained positive control samples (complete reaction mix, no inhibitor test compound), negative control samples (reaction mix without TPH1 enzyme), and an additional 5-HTP control (reaction mix without TPH1 enzyme, spiked with 1.5 or 2.25 μM 5-HTP (Sigma-Aldrich)) to measure the progression of the enzymatic reaction. Samples were measured in technical triplicates. The TPH1 assay was performed at room temperature (RT) for 10 min. The formation of the reaction product (5-HTP; 5-hydroxytryptophan) was continuously monitored in 30 s intervals by fluorescence measurements (ex.300 nm/em.330 nm) for 2.5 min using a Tecan Infinite 200 plate reader.

### TPH2 Activity Assay

The TPH2 enzymatic activity assay was performed using a 96-well plate format (Greiner). The TPH2 reaction mixture (total volume of 200 μL) contained: 50 mM MES buffer (pH 7.0) (Sigma-Aldrich), 50 μM FAS (Sigma-Aldrich), 7.5 μM BH_4_ (Cayman Chemicals), 0.05 mg/mL catalase (Sigma-Aldrich), 5 mM DTT (Sigma-Aldrich), and 50 nM human recombinant TPH2 enzyme. To initiate the reaction, l-Trp (Sigma-Aldrich) was added to a final concentration of 30 μM. The assay contained positive control samples (complete reaction mix, no inhibitor test compound), negative control samples (reaction mix without TPH2 enzyme), and an additional 5-HTP control (reaction mix without TPH2 enzyme, spiked with 3 or 4.5 μM 5-HTP (Sigma-Aldrich)) to measure the progression of the enzymatic reaction. All samples were measured in technical triplicates. The TPH2 assay was performed at RT for 10 min. The formation of the reaction product (5-HTP; 5-hydroxytryptophan) was continuously monitored at 30 s intervals by fluorescence measurements (ex.300 nm/em.330 nm) for 5 min using a Tecan Infinite 200 plate reader.

### TH Activity Assay

The TH enzymatic activity assay was performed using a 96-well plate format (Greiner). The TH reaction mixture (total volume of 200 μL) contained 50 mM HEPES buffer (pH 7.0) (Carl Roth), 50 μM FAS (Sigma-Aldrich), 5 μM BH_4_ (Cayman Chemicals), 0.05 mg/mL catalase (Sigma-Aldrich), 5 mM DTT (Sigma-Aldrich), and 500 nM human recombinant PAH enzyme. To initiate the reaction, l-Tyr (Fluka) was added (final concentration, 30 μM). The assay contained positive control samples (complete reaction mix, no inhibitor test compound), negative control samples (reaction mix without BH_4_), and an additional l-Tyr control (reaction mix without BH_4_, spiked with 25.5 μM or 27 μM l-Tyr) to measure the progression of the enzymatic reaction. All samples were measured in technical triplicates. The TH assay was performed at RT for 30 min. Depletion of the reaction substrate (l-Tyr) was continuously monitored at 240 s intervals by fluorescence measurements (ex.280 nm/em.306 nm) for 12 min using a Tecan Infinite 200 plate reader.

### PAH Activity Assay

The PAH enzymatic activity assay was performed using a 96-well plate format (Greiner). The PAH reaction mixture (total volume of 200 μL) contained 50 mM MES buffer (pH 7.0) (Sigma-Aldrich), 50 μM FAS (Sigma-Aldrich), 10 μM BH_4_ (Cayman Chemicals), 0.05 mg/mL catalase (Sigma-Aldrich), 5 mM DTT (Sigma-Aldrich), and 200 nM human recombinant PAH enzyme. To initiate the reaction, l-Phe (Sigma-Aldrich) was added at a final concentration of 175 μM. The assay contained positive control samples (complete reaction mix, no inhibitor test compound), negative control samples (reaction mix without PAH enzyme), and an additional l-Tyr control samples (reaction mix without PAH enzyme, spiked with 17.5 or 26.25 μM l-Tyr (Fluka)) to measure the progression of the enzymatic reaction. All samples were measured in technical triplicates. The PAH assay was performed at RT for 30 min. The formation of the reaction product (l-Tyr; l-tyrosine) was continuously monitored at 120 s intervals by fluorescence measurements (ex.280 nm/em.306 nm) for 12 min using a Tecan Infinite 200 plate reader.

### AAAH Assay Data Analysis and IC_50_ Determination

Negative control values (no enzyme or no substrate) were deducted from the positive control (full enzyme activity) and test compound values. Subsequently, the data were normalized (Pos. Ctr. = 100% of the enzyme activity). The results were further analyzed with GraphPad Prism 9 using the nonlinear regression model:

log(inhibitor) vs.⁢ response−variableslope(fourparameters)

which fits the following equation to the data:

$$Y=\mathrm{Bottom}+\left(\mathrm{Top}-\mathrm{Bottom}\right)/(1+10^{\left(\left({\log\;\mathrm{IC}}_{50}-X\right)\times \mathrm{HillSlope}\right)})$$

The IC_50_ values were calculated from three independent experiments and are given as the mean ± SD.

### Broad Selectivity Profiling

TPT-004 was evaluated at a concentration of 10 μM in a total of 97 binding, enzyme, and uptake assays (Diversity Panel) by Eurofins Cerep (Celle l’Evescault, France). Compound binding was calculated as % inhibition of the binding of a ligand specific to each target. The compound enzyme inhibition effect was calculated as percentage inhibition of the control enzyme activity. Results showing an inhibition (or stimulation for assays run under basal conditions) of >50% are considered to represent significant effects of the test compounds. Results showing an inhibition (or stimulation) between 25% and 50% are indicative of weak to moderate effects. Results showing an inhibition (or stimulation) lower than 25% are not considered significant and are mostly attributable to variability in the signal around the control level. Low to moderate negative values have no real meaning and are attributable to the variability of the signal around the control level. In each experiment, and if applicable, the respective reference compound was tested concurrently with TPT-004, and the data were compared with historical values determined at Eurofins. The experiment was accepted in accordance with the Eurofins validation Standard Operating Procedure.

#### Binding Assays

The results are expressed as a percent of control-specific binding:

$$\frac{\mathrm{measured specific binding}}{\mathrm{control specific binding}}\times 100$$

and as a percent inhibition of control-specific binding:

$$\mathrm{100}-(\frac{\mathrm{measured specific binding}}{\mathrm{control specific binding}}\times 100)$$

obtained in the presence of TPT-004.

The IC_50_ values (concentration causing a half-maximal inhibition of control-specific binding) and Hill coefficients (nH) were determined by nonlinear regression analysis of the competition curves generated with mean replicate values using Hill equation curve fitting:

$$Y=D+\left[\frac{A-D}{1+{\left(C/C_{50}\right)}^{\mathrm{nH}}}\right]$$

where Y = specific binding, A = left asymptote of the curve, D = right asymptote of the curve, C = compound concentration, *C*_50_ = IC_50_, and nH = slope factor. This analysis was performed using software developed at Cerep (Hill software) and validated by comparison with data generated by the commercial software SigmaPlot 4.0 for Windows (© 1997 by SPSS Inc.).

The specificity ratios were calculated by dividing the IC_50_ value for the off-target region by the IC_50_ value for the primary target.

#### Enzyme and Uptake Assays

The results are expressed as a percent of control-specific binding:

$$\frac{\mathrm{measured specific binding}}{\mathrm{control specific binding}}\times 100$$

and as a percent inhibition of control-specific binding:

$$\mathrm{100}-\left(\frac{\mathrm{measured specific binding}}{\mathrm{control specific binding}}\times 100\right)$$

obtained in the presence of TPT-004.

The IC_50_ values (concentration causing a half-maximal inhibition of control-specific activity), EC_50_ values (concentration producing a half-maximal increase in control basal activity), and Hill coefficients (nH) were determined by nonlinear regression analysis of the inhibition/concentration–response curves generated with mean replicate values using Hill equation curve fitting:

$$Y=D+\left[\frac{A-D}{1+{\left(C/C_{50}\right)}^{\mathrm{nH}}}\right]$$

where *Y* = specific activity, *A* = left asymptote of the curve, *D* = right asymptote of the curve, *C* = compound concentration, *C*_50_ = IC_50_ or EC_50_, and nH = slope factor. This analysis was performed using software developed at Cerep (Hill software) and validated by comparison with data generated by the commercial software SigmaPlot 4.0 for Windows (© 1997 by SPSS Inc.).

### Dose Range-Finding (DRF) Study in Rats

The in-life phase of the experiment was performed at Pharmacelsus GmbH (Saarbrücken, Germany) and enrolled 24 Sprague–Dawley rats (12 males and 12 females) provided by Janvier Laboratories (France); the animals were 7 weeks old at delivery. Inclusion criteria (sex, appropriate body weight, and absence of behavioral changes) were established prior to the study’s commencement. The animals were housed in a temperature-controlled room (20–24 °C) and maintained in a 12 h light/12 h dark cycle. Before the start of the study, the animals underwent a six-day acclimation period. Food and water were available *ad libitum* throughout the duration of the study.

TPT-004 was formulated in 0.5% (w/v) CMC-Na in water and administered by oral gavage (PO) twice daily (b.i.d.) with a 12 h interval to the treatment groups (3 male and 3 female Sprague–Dawley rats each) for 8 days (15 successive applications). Rats were dosed with 10, 45, and 200 mg/kg, i.e., daily doses of 20, 90, and 400 mg/kg. In addition, a control group was dosed at the same frequency as the vehicle (0.5% CMC-Na).

The rats were observed for clinical signs daily; their body weight was monitored daily, and their food consumption was recorded twice during the experiment. Blood samples were collected as described below. The animal sacrifice (exsanguination) was performed on day 8. During the necropsy, the selected organs were collected, weighed, and shipped to a third-party laboratory for histopathological examination (as described below).

The study director was cognizant of group allocation throughout the experiment. The blinding procedure was extended to veterinary and laboratory technicians responsible for treatment administration and terminal surgery. All experimental procedures were approved by and conducted in accordance with the regulations of the local Animal Welfare authorities (Landesamt für Gesundheit und Verbraucherschutz, Abteilung Lebensmittel- und Veterinärwesen, Saarbrücken) under case number GB 4–2.4.2.2.–28–2021.

### Blood Sampling

Blood samples for quantification of TPT-004 by LC-MS were collected on days 1 and 8 (after 15 doses) at Tmax (0.5 h post-dose). A blood sample of 100 μL was obtained from each rat by puncturing one of the lateral tail veins under short isoflurane anesthesia. Blood was collected in tubes containing lithium heparin and gently mixed. After centrifugation (10 min at 3000*g*, 4 °C), plasma was separated and stored at −20 °C until LC-MS analysis. In addition, on day 8, the rats were exsanguinated by cardiac puncture under isoflurane anesthesia. A volume of 100 μL of blood (K3-EDTA) was used for immediate cell blood count, while the rest of the blood was mixed with lithium heparin and centrifuged to generate plasma for clinical chemistry analysis.

### Histopathology

The histological evaluation was performed by AnaPath Services GmbH (Liestal, Switzerland). The samples from the organs—liver, kidney, lung, heart, brain, femur, and ovary—were fixed in 4% neutral-buffered formaldehyde. The testes were fixed by using the Davidson solution. All tissues were trimmed, processed, and embedded in paraffin wax. Samples from the femur were processed and embedded in paraffin after decalcification. Blocks were cut at an approximate thickness of 2–4 μm and stained with hematoxylin and eosin (HE) according to AnaPath Services GmbH SOP’s. The slides were checked under the microscope for a quality check before examination under a light microscope by the study pathologist.

## Supplementary Material





## Notes

Molecular formula strings (CSV)
Table S1: *K*_m_ values for substrate and cosubstrate in TPH1, TPH2, PAH, and TH assay; Table S2: enzymatic activity of TPT-004, LP778902, and LX1606 in TPH1 assay; Table S3: enzymatic activity of TPT-004, LP778902, and LX1606 in TPH2 assay; Table S4: enzymatic activity of TPT-004, LP778902, and LX1606 in PAH assay; Table S5: enzymatic activity of TPT-004, LP778902, and LX1606 in TH assay; Table S6: hematology parameters in Sprague–Dawley rats; Table S7: clinical chemistry parameters in Sprague–Dawley rats; Table S8: organ weight in Sprague–Dawley rats; Table S9: data collection and structure refinement statistics; Figure S1: superimposition of TPH1 and TPH2 complex crystal structures; Figure S2: H&E staining of a liver of a Sprague–Dawley rat in hepatocellular centrilobular hypertrophy (PDF)

## Abbrevations

AAAH: aromatic amino acid hydroxylase
AGO RL: agonist radio ligand
ANT RL: antagonist radio ligand
BH_4_: tetrahydrobiopterin
BID: bis in die
CI: confidence interval
CMC-Na: sodium carboxymethyl cellulose
DTT: dithiothreitol
DRF: dose range finding
EDTA: ethylenediaminetetraacetic acid
EMA: European Medicines Agency
FAS: ferrous ammonium sulfate
HPLC: high-performance liquid chromatography
HTS: high-throughput screening
5-HTP: 5-hydroxytryptophan
HEPES: 4-(2-hydroxyethyl)-1-piperazineethanesulfonic acid
IC_50_: half-maximal inhibitory concentration
IPTG: isopropyl-β-d-thiogalactopyranoside
K_m_: Michaelis constant, the substrate concentration needed for half-maximum velocity
MASLD: metabolic dysfunction-associated steatotic liver disease
MBP: maltose-binding protein
MES: 2-(*N*-morpholino)­ethanesulfonic acid
PAH: phenylalanine hydroxylase
PDB: protein database
PMSF: phenylmethylsulfonyl fluoride
PLT: number of platelets
PO: per os
RDW: red cell distribution width
RT: room temperature
SAR: structure–activity relationship
SD: standard deviation
5-HT: serotonin
TB: terrific broth
TPH1: tryptophan hydroxylase 1
TPH2: tryptophan hydroxylase 2
TH: tyrosine hydroxylase
