# Supplementary material for: Selectivity and Safety Characterization of a Xanthine–Imidazothiazole Lead Structure: a Novel Tryptophan Hydroxylase Inhibitor of Peripheral Serotonin Synthesis
Source: ACS Pharmacol Transl Sci. 2025 May 19;8(6):1678–93. doi: 10.1021/acsptsci.5c00043 (PMC12171880; doi:10.1021/acsptsci.5c00043)
Supplement: Supplementary file 2 [file pt5c00043_si_002.pdf]

Selectivity and safety characterization of a xanthine-imidazothiazole lead structure, a novel tryptophan hydroxylase inhibitor of peripheral serotonin synthesis

Radoslaw Wesolowski[1,2,#], Anja Schütz[2,#], Michael Lisurek[3], Marc Nazaré[3], Udo Heinemann[2], Dirk Pleimes[1], Michael Bader[1,2,4,5,6,7*], Edgar Specker[1,3*]

[#]these authors contributed equally

[1]Trypto Therapeutics GmbH, Robert-Rössle Str. 10, 13125 Berlin, Germany. [2]Max-Delbrück-Center for Molecular Medicine in the Helmholtz Association (MDC), Robert-Rössle-Str. 10, 13125 Berlin-Buch, Germany. [3]Chemical Biology Platform, Leibniz-Forschungsinstitut für Molekulare Pharmakologie (FMP), Robert-Rössle-Str. 10, 13125 Berlin-Buch, Germany. [4]German Center for Cardiovascular Research (DZHK), Partner Site Berlin, Potsdamer Str. 58, 10785 Berlin, Germany. [5]Charité - Universitätsmedizin Berlin, Charitéplatz 1, 10117 Berlin, Germany. [6]Experimental and Clinical Research Center, a cooperation between the Max-Delbrück-Center for Molecular Medicine in the Helmholtz Association and the Charité - Universitätsmedizin Berlin, Lindenberger Weg 80, 13125 Berlin-Buch, Germany. [7]University of Lübeck, Institute for Biology, Ratzeburger Allee 160, 23562 Lübeck, Germany

*corresponding authors: mbader@mdc-berlin.de and specker@fmp-berlin.de

Supporting Information

Table of Contents

Supporting Information Table S1. K_m values for substrate and cosubstrate in TPH1, TPH2, PAH and TH assay....S3

Supporting Information Table S2. Enzymatic activity of TPT-004, LP778902 and LX1606 in TPH1 assay....S3

Supporting Information Table S3. Enzymatic activity of TPT-004, LP778902 and LX1606 in TPH2 assay....S4

Supporting Information Table S4. Enzymatic activity of TPT-004, LP778902 and LX1606 in PAH assay....S4

Supporting Information Table S5. Enzymatic activity of TPT-004, LP778902 and LX1606 in TH assay....S5

Supporting Information Table S6. Hematology parameters in Sprague-Dawley rats....S5

Supporting Information Table S7. Clinical chemistry parameters in Sprague-Dawley rats....S6

Supporting Information Table S8. Organ weight in Sprague-Dawley rats....S6

Supporting Information Table S9. Data collection and structure refinement statistics.S7

Supporting Information Figure S1. Superimposition of TPH1 and TPH2 complex crystal structures....S8

Supporting Information Figure S2. H&E staining of a liver of a Sprague-Dawley rat in hepatocellular centrilobular hypertrophy S9

Supporting Information Table S1. K_m values for substrate and cosubstrate in TPH1, TPH2, PAH and TH assay

	Determined K_m for main substrate	Main substrate concentration used in the assay	Determined K_m for BH_4	BH_4 concentration used in the assay
**TPH1 assay**	L-Trp: 13.18 µM (95% CI 11.05 - 15.83 µM)	L-Trp: 15 µM	BH_4: 17.68 µM (95% CI 12.55 - 25.96 µM)	BH_4: 20 µM
**TPH2 assay**	L-Trp: 30.72 µM (95% CI 27.35 - 34.66 µM)	L-Trp: 30 µM	BH_4: 6.57 µM (95% CI 5.81 - 7.45 µM)	BH_4: 7.5 µM
**PAH assay**	L-Phe: 171.7 µM (95% CI 150.6 - 196.0 µM)	L-Phe: 175 µM	BH_4: 13.73 µM (95% CI 10.46 - 18.52 µM)	BH_4: 10 µM
**TH assay**	L-Tyr 29.07 µM (95% CI 19.30 - 46.18 µM)	L-Tyr: 30 µM	BH_4: 1.96 µM (95% CI 1.13 - 3.45 µM)	BH_4: 5 µM

Supplementary Table S1. K_m values for the substrate (L-Trp or L-Phe or L-Tyr) and cosubstrate (BH_4) were determined for AAAH family members (TPH1, TPH2, PAH, TH). The table includes the final concentrations selected for the subsequent enzymatic assays.

Supporting Information Table S2. Enzymatic activity of TPT-004, LP778902 and LX1606 in TPH1 assay

Drug concentration	Log [M] concentration	TPT-004		LP778902		LX1606	
		Mean TPH1 activity [%]	SD	Mean TPH1 activity [%]	SD	Mean TPH1 activity [%]	SD
10 µM	-5	3.21	2.57	5.34	4.42	6.31	2.84
3 µM	-5.5	3.64	2.04	4.78	4.11	4.22	3.10
1 µM	-6	3.62	1.06	12.20	14.79	80.38	9.14
300 nM	-6.5	7.59	1.14	91.38	9.04	94.76	6.68
100 nM	-7	21.29	2.28	100.00	8.14	93.76	5.77
30 nM	-7.5	52.25	5.20	101.72	12.43	103.86	9.64
10 nM	-8	77.11	4.65	100.85	7.91	101.82	8.54
3 nM	-8.5	99.51	5.73	99.75	5.63	105.40	2.70

Supplementary Table S2. TPT-004, LP778902, and LX1606 effect on TPH1 enzymatic activity. The mean TPH1 enzymatic activity ± SD was calculated from five independent experiments (TPT-004 and LP778902) or four independent experiments (LX1606). The relative activity of TPH1 was determined based on the positive control (assumed 100% enzymatic activity) and negative control (assumed 0% enzymatic activity) samples. Each drug concentration was tested in three technical replicates per experiment.

Supporting Information Table S3. Enzymatic activity of TPT-004, LP778902 and LX1606 in TPH2 assay

Drug concentration	Log [M] concentration	TPT-004		LP778902		LX1606	
		Mean TPH2 activity [%]	SD	Mean TPH2 activity [%]	SD	Mean TPH2 activity [%]	SD
10 µM	-5	1.24	1.49	3.17	1.86	5.40	2.05
3 µM	-5.5	1.07	1.49	3.90	1.06	4.11	1.53
1 µM	-6	0.69	0.49	10.61	9.60	66.80	16.89
300 nM	-6.5	1.60	1.18	92.18	4.23	97.78	4.23
100 nM	-7	5.40	0.38	97.93	8.01	95.32	6.59
30 nM	-7.5	38.03	2.88	99.88	8.55	101.26	4.71
10 nM	-8	73.97	3.55	100.78	5.68	97.14	7.66
3 nM	-8.5	100.37	3.40	99.93	5.53	107.79	3.38

Supplementary Table S3. TPT-004, LP778902, and LX1606 effect on TPH2 enzymatic activity. The mean TPH1 enzymatic activity ± SD was calculated from five independent experiments (TPT-004 and LP778902) or six independent experiments (LX1606). The relative activity of TPH2 was determined based on the positive control (assumed 100% enzymatic activity) and negative control (assumed 0% enzymatic activity) samples. Each drug concentration was tested in three technical replicates per experiment.

Supporting Information Table S4. Enzymatic activity of TPT-004, LP778902 and LX1606 in PAH assay

Drug concentration	Log [M] concentration	TPT-004		LP778902		LX1606	
		Mean PAH activity [%]	SD	Mean PAH activity [%]	SD	Mean PAH activity [%]	SD
30 µM	-4.5	2.49	1.66	7.18	2.75	33.74	2.33
10 µM	-5	4.37	2.13	18.22	4.42	37.02	2.82
3 µM	-5.5	9.81	2.94	42.58	4.89	64.20	4.38
1 µM	-6	25.53	4.27	79.01	9.48	91.16	7.67
300 nM	-6.5	58.10	3.81	92.85	2.53	94.55	5.14
100 nM	-7	82.79	1.70	95.74	3.42	93.78	4.41
30 nM	-7.5	91.12	2.56	96.34	2.77	97.35	3.97
10 nM	-8	94.75	3.00	99.68	3.34	96.86	5.33

Supplementary Table S4. TPT-004, LP778902, and LX1606 effect on PAH enzymatic activity. The mean TPH1 enzymatic activity ± SD was calculated from six independent experiments (TPT-004) or five independent experiments (LP778902, LX1606). The relative activity of PAH was determined based on the positive control (assumed 100% enzymatic activity) and negative control (assumed 0% enzymatic activity) samples. Each drug concentration was tested in three technical replicates per experiment.

Supporting Information Table S5. Enzymatic activity of TPT-004, LP778902 and LX1606 in TH assay

Drug concentration	Log [M] concentration	TPT-004		LP778902		LX1606	
		Mean TH activity [%]	SD	Mean TH activity [%]	SD	Mean TH activity [%]	SD
30 µM	-4.5	-6.30	3.07	14.34	11.10	71.72	8.83
10 µM	-5	6.59	9.86	43.11	13.56	87.53	6.93
3 µM	-5.5	28.97	8.03	79.57	6.76	96.83	6.90
1 µM	-6	58.14	5.27	89.03	5.03	96.27	6.29
300 nM	-6.5	86.81	6.42	96.86	10.52	91.31	10.25
100 nM	-7	95.57	7.80	91.73	7.58	94.52	10.03
30 nM	-7.5	90.14	7.93	96.35	6.17	93.57	11.45
10 nM	-8	92.60	7.13	91.23	12.97	94.16	11.02

Supplementary Table S5. TPT-004, LP778902, and LX1606 effect on TH enzymatic activity. The mean TPH1 enzymatic activity ± SD was calculated from five independent experiments. The relative activity of TH was determined based on the positive control (assumed 100% enzymatic activity) and negative control (assumed 0% enzymatic activity) samples. Each drug concentration was tested in three technical replicates per experiment.

Supporting Information Table S6. Hematology parameters in Sprague-Dawley rats

	Vehicle		TPT-004 10 mg/kg PO BID		TPT-004 45 mg/kg PO BID		TPT-004 200 mg/kg PO BID	
	Mean	SD	Mean	SD	Mean	SD	Mean	SD
LEU (x10^9/l)	12.51	8.62	13.08	4.01	17.48	1.29	15.34	3.65
LYM (x10^9/l)	10.71	7.58	10.91	3.64	14.46	1.11	12.63	2.78
MON (x10^9/l)	0.45	0.50	0.58	0.44	1.07	0.21	0.74	0.21
NEU (x10^9/l)	1.36	0.95	1.59	0.51	1.95	0.64	1.97	0.79
LYM (%)	84.5	8.7	82.8	4.0	82.7	2.5	82.9	3.8
MON (%)	2.9	2.6	4.0	2.8	6.2	1.3	4.8	0.8
NEU (%)	12.7	8.4	13.3	6.0	11.1	3.5	12.3	3.5
ERY (x10^{12}/l)	7.01	1.88	8.15	0.47	8.12	0.57	8.03	0.55
HGB (g/dl)	12.2	3.2	13.9	0.5	14.0	0.5	13.1	0.5
HCT (%)	43.45	10.66	49.64	1.96	48.67	2.33	45.70	1.72
MCV (fl)	63	3	61	2	60	2	57	4
MCH (pg)	17.5	0.7	17.1	0.6	17.3	0.8	16.4	1.3
MCHC (g/dl)	28.0	0.7	28.0	0.2	28.8	0.8	28.7	0.4
RDWc (%)	16.4	0.8	16.4	0.4	17.2	0.5	19.3	1.6
RDWs (fl)	38.4	2.0	37.6	1.9	38.7	2.0	40.8	1.6
PLT (x10^9/l)	91	91	169	152	104	39	179	146
MPV (fl)	8.3	1.0	8.2	1.5	8.3	0.6	8.3	1.0
PCT (%)	0.10	0.08	0.15	0.09	0.08	0.03	0.14	0.10
PDWc (%)	34.9	1.7	33.7	5.1	36.1	1.0	35.8	3.4
PDWs (fl)	12.7	1.6	12.3	5.3	13.9	1.4	13.7	3.2

Supplementary Table 6. Hematology parameters in Sprague-Dawley rats (n=6 per group) were assessed on day 8. Values are expressed as mean ± SD.

Supporting Information Table S7. Clinical chemistry parameters in Sprague-Dawley rats

	Vehicle		TPT-004 10 mg/kg PO BID		TPT-004 45 mg/kg PO BID		TPT-004 200 mg/kg PO BID	
	Mean	SD	Mean	SD	Mean	SD	Mean	SD
Albumin (g/L)	30	1	31	1	30	1	30	1
Globulin (g/L)	23	1	23	1	23	1	23	1
Albumin/ globulin ratio	1.27	0.05	1.33	0.07	1.34	0.06	1.32	0.03
Alkaline phosphatase (U/L)	470	173	402	83	434	145	313	146
ALT (GPT) (U/L)	58	9	49	7	52	7	67	10
Bile acids (µmol/L)	6.6	3.6	6.3	4.6	16.6	13.9	20.4	13.4
Bilirubin total (µmol/L)	1.7	0.0	1.7	0.0	1.7	0.0	1.7	0.0
Calcium (mmol/L)	2.7	0.1	2.6	0.1	2.7	0.1	2.6	0.1
Chloride (mmol/L)	100	1	101	2	101	1	101	2
Cholesterol (mmol/L)	2.4	0.3	2.25	0.3	2.5	0.3	2.6	0.4
Creatine kinase (U/L)	285	119	406	80	415	159	321	79
Creatinine (µmol/L)	24	4	24	3	26	5	24	2
GLDH (U/L)	6	1	7	1	6	1	7	1
Glucose (mmol/L)	12.7	2.7	11.2	0.8	12.3	1.9	13.5	2.2
Inorganic phosphate (mmol/L)	2.4	0.2	2.3	0.2	2.4	0.1	2.4	0.4
Potassium (mmol/L)	5.1	0.6	4.7	0.4	4.7	0.5	4.9	0.3
Sodium (mmol/L)	142	1	143	2	142	2	143	1
Total protein (g/L)	53	2	54	2	53	2	53	2
Triglycerides (mmol/L)	1.4	0.8	1.2	0.7	1.7	0.9	1.4	0.5
Urea (BUN) (mmol/L)	6.4	0.8	6.4	0.7	6.0	0.6	7.0	1.2

Supplementary Table S7. Clinical chemistry parameters in Sprague-Dawley rats (n=6 per group) were assessed on day 8. Values are expressed as mean ± SD.

Supporting Information Table S8. Organ weight in Sprague-Dawley rats

	Vehicle		TPT-004 10 mg/kg PO BID		TPT-004 45 mg/kg PO BID		TPT-004 200 mg/kg PO BID	
	Mean	SD	Mean	SD	Mean	SD	Mean	SD
Brain	6.3	1.2	6.8	1.6	6.5	1.2	6.7	1.3
Heart	3.6	0.2	3.5	0.3	3.6	0.2	3.6	0.3
Liver	41.2	2.2	39.4	3.5	42.1	1.7	45.2	1.2
Kidney	3.6	0.2	3.8	0.4	3.6	0.2	4.0	0.3
Lung	5.0	0.8	5.3	0.7	5.2	0.9	5.2	0.7
Spleen	2.8	0.2	2.9	0.2	2.8	0.3	2.8	0.5
Ovary (n=3 per group)	0.62	0.09	0.72	0.15	0.55	0.15	0.65	0.04
Testis (n=3 per group)	4.7	2.7	8.7	0.5	8.1	0.7	8.4	0.2

Supplementary Table S8. Organ weight (g/kg body weight) in Sprague-Dawley rats (n=6 per group; n=3 males and n=3 females) was assessed on day 8. Values are expressed as mean ± SD.

Supporting Information Table S9. Data collection and structure refinement statistics

	hTPH2<br>AG-01-128	hTPH2<br>KM-06-098
PDB ID code	9HB7	9HB8
**Data collection**		
Space group	$P2_12_12$	$P2_12_12$
Cell dimensions		
a, *b*, *c* (Å)	101.14, 94.65, 88.74	100.51, 94.11, 87.92
α, β, γ (°)	90, 90, 90	90, 90, 90
Resolution (Å)	47.32-2.96 (3.13-2.96)*	47.05-2.90 (3.07-2.90)
R_{merge} (%)	10.01 (149.7)	9.04 (150.7)
$< I / \sigma(I) >$	16.65 (1.39)	15.54 (1.30)
Completeness (%)	99.65 (98.39)	99.47 (97.73)
Redundancy	5.8 (6.0)	6.2 (6.4)
**Refinement**		
Resolution (Å)	2.96	2.90
No. unique reflections	18356	19065
R_{work} / R_{free} (%)	20.48 / 24.39	20.93 / 24.86
No. non-hydrogen atoms	5498	5565
Protein	5434	5489
Ligands	62	74
Water	2	2
Average B-factor (Å²)		
Overall	101.2	106.7
Protein	100.9	106.6
Ligands	121.1	116.0
Water	85.3	73.9
R.m.s deviations		
Bond lengths (Å)	0.005	0.005
Bond angles (º)	1.05	1.15

*Values in parenthesis are for highest resolution shell.
One single crystal was used to collect a complete dataset for each structure.

Supplementary Table S9. Data collection and refinement statistics for the human TPH2 complex crystal structures.

Supporting Information Figure S1. Superimposition of TPH1 and TPH2 complex crystal structures

The two new TPH2 complex crystal structures were determined to provide a structural basis for comparing TPH1 and TPH2 inhibitor binding at atomic level. Since TPH1 and TPH2 share high sequence similarity and nearly identical active site architectures, analyzing these structures allows us to confirm whether the key binding interactions observed in TPH1 are also present in TPH2. This is crucial for understanding inhibitor selectivity, as any structural differences between TPH1 and TPH2 that influence inhibitor binding could help explain why an inhibitor preferentially targets TPH1 over TPH2. By systematically evaluating these structures, we can determine whether selectivity arises from subtle variations in binding pocket geometry, subpocket composition, or dynamic properties that may not be evident from sequence alignments alone.

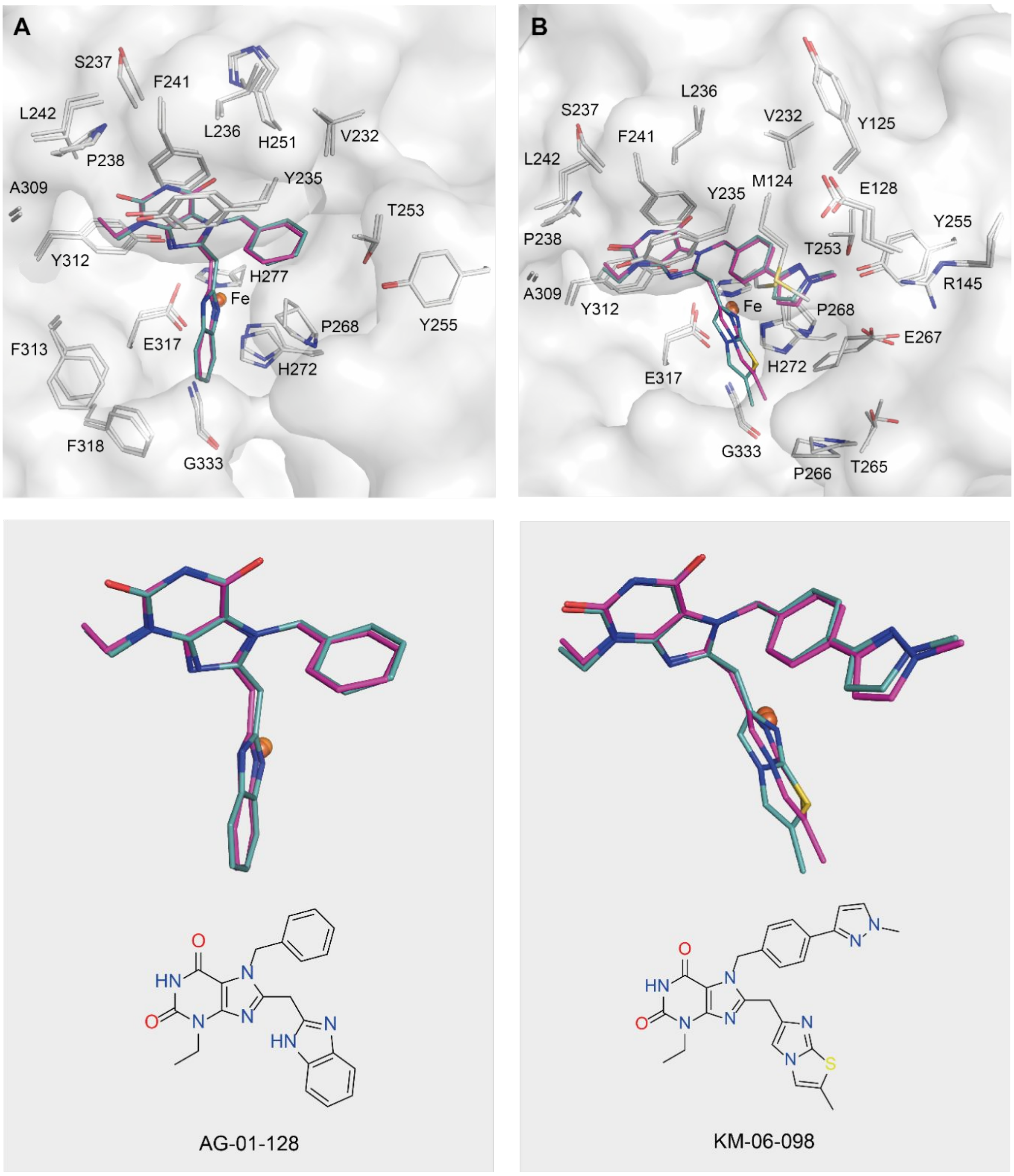

Supplementary Figure S1. Superimposition of human TPH1 and TPH2 complex crystal structures. Residues within 5 Å of the bound inhibitor (C: magenta/teal for TPH1/2, O: red, N: blue) are shown in stick model (C: gray, O: red, N: blue, S: yellow) and labelled accordingly (for clarity, only TPH1 residues are labeled; shown TPH1 and TPH2 active site residues are fully conserved; TPH2 residues are offset by +46 relative to the TPH1 numbering). The iron atom is displayed as orange sphere. The gray surface representation refers to TPH1. (A) Complex with AG-01-128 (TPH1: inhibitor **29** in [19], PDB ID code 7ZIH; TPH2: PDB ID code 9HB7, this study). Both structures superimpose with an r.m.s.d. value of 0.63 Å. (B) Complex with KM-06-098 (TPH1: inhibitor **16** in [20], PDB ID code 8CJK; TPH2: PDB ID code 9HB8, this study). Both structures superimpose with an r.m.s.d. value of 0.57 Å.

Supporting Information Figure S2. H&E staining of a liver of a Sprague-Dawley rat in hepatocellular centrilobular hypertrophy

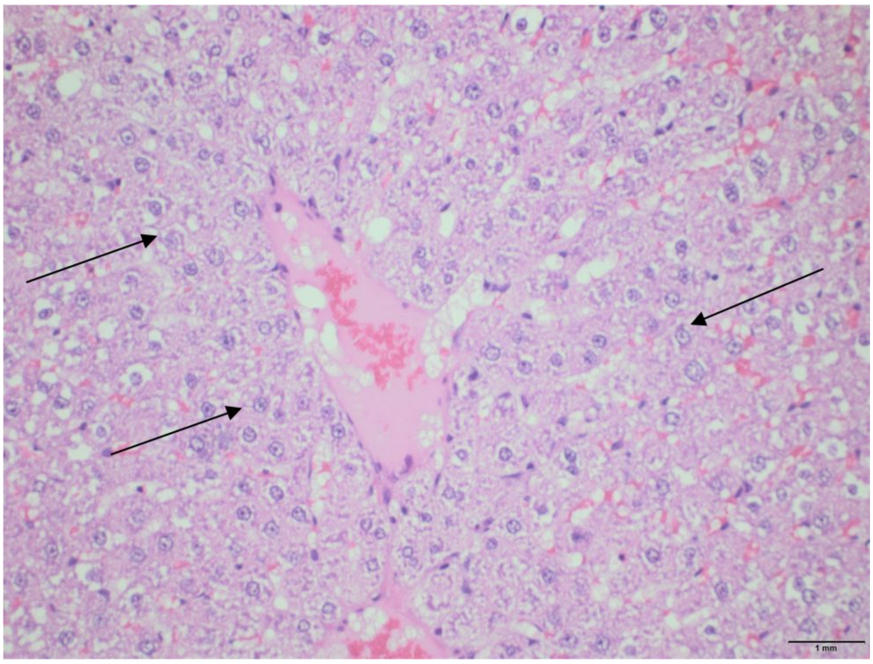

Supplementary Figure S2. Example of hepatocellular centrilobular hypertrophy in a male Sprague-Dawley rat treated with TPT-004 at 200 mg/kg BID for 8 days. H&E staining of the liver sections, original magnification × 20. Multifocally hepatocytes from centrilobular areas showed minimal hypertrophy (arrows).
